# C/D box snoRNA SNORD113-6 guides 2′-O-methylation and protects against site-specific fragmentation of tRNA^Leu^(TAA) in vascular remodeling

**DOI:** 10.1016/j.omtn.2022.09.011

**Published:** 2022-09-17

**Authors:** Eva van Ingen, Pleun A.M. Engbers, Tamar Woudenberg, M. Leontien van der Bent, Hailiang Mei, Johann Wojta, Paul H.A. Quax, A. Yaël Nossent

**Affiliations:** 1 Department of Surgery, Leiden University Medical Center, PO Box 9600, 2300 Leiden, the Netherlands; 2 Einthoven Laboratory for Experimental Vascular Medicine, Leiden University Medical Center, Leiden, the Netherlands; 3 Department of Biomedical Data Sciences, Leiden University Medical Center, Leiden, the Netherlands; 4 Department of Internal Medicine II, Medical University of Vienna, Vienna, Austria; 5 Department of Laboratory Medicine, Medical University of Vienna, Vienna, Austria; 6 Ludwig Boltzmann Institute for Cardiovascular Research, Vienna, Austria

**Keywords:** MT: RNA/DNA Editing, tRNA-derived fragments, tRF, C/D box small nucleolar RNAs, orphan, 14q32 locus, DLK1-DIO3 locus, cardiovascular disease, vascular remodeling

## Abstract

C/D box small nucleolar RNAs (snoRNAs) of the DLK1-DIO3 locus are associated with vascular remodeling and cardiovascular disease. None of these snoRNAs has any known targets yet except for one, AF357425/SNORD113-6. We previously showed that this snoRNA targets mRNAs of the integrin signaling pathway and affects arterial fibroblast function. Here, we aimed to identify whether AF357425/SNORD113-6 can also target small RNAs. We overexpressed or inhibited AF357425 in murine fibroblasts and performed small RNA sequencing. Expression of transfer (t)RNA fragments (tRFs) was predominantly regulated. Compared with overexpression, AF357425 knockdown led to an overall decrease in tRFs but with an enrichment in smaller tRFs (<30 nucleotides). We focused on tRNA leucine anti-codon TAA (tRNA^Leu^(TAA)), which has a conserved predicted binding site for AF357425/SNORD113-6. Adjacent to this site, the tRNA is cleaved to form tRF^Leu 47–64^ in both primary murine and human fibroblasts and in intact human arteries. We show that AF357425/SNORD113-6 methylates tRNA^Leu^(TAA) and thereby prevents the formation of tRF^Leu 47–64^. Exposing fibroblasts to oxidative or hypoxic stress increased AF357425/SNORD113-6 and tRNA^Leu^(TAA) expression, but AF357425/SNORD113-6 knockdown did not increase tRF^Leu 47–64^ formation under stress even further. Thus, independent of cellular stress, AF357425/SNORD113-6 protects against site-specific fragmentation of tRNA^Leu^(TAA) via 2′O-ribose-methylation.

## Graphical abstract

**Figure:**
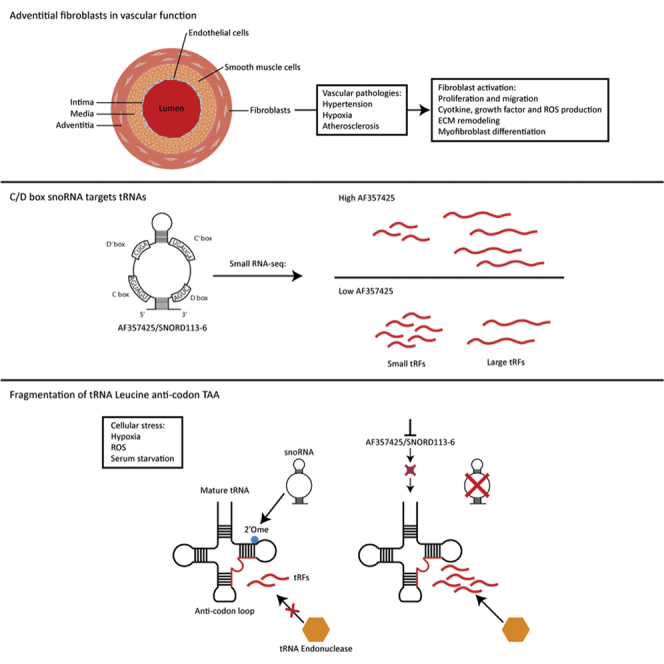

## Teaser

The vasoactive 14q32 snoRNA AF357425/SNORD113-6 targets tRNAs to induce 2′O-ribose-methylation. For tRNA^Leu^(TAA), methylation protects the tRNA from site-specific cleavage into tRF^Leu 47–64^ in murine and human primary fibroblasts. tRNA fragmentation is induced under cellular stress. Upregulation of SNORD113-6 in human arteries is associated with decreased tRNA fragmentation under ischemia *ex vivo*.

## Introduction

Vascular remodeling is the collective name for both adaptive and maladaptive changes to the vessel wall. This includes processes like angiogenesis and arteriogenesis on the one hand and atherosclerosis and aneurysm formation on the other.^1^ Vascular remodeling is the predominant underlying cause of most cardiovascular disease. All layers of the arterial wall, i.e., the tunica intima made up of endothelial cells, the tunica media made up of smooth muscle cells, and the tunica adventitia made up of predominantly fibroblasts, play their own role in vascular remodeling, but the role of the adventitial fibroblasts is often underestimated.^2^ Although cardiovascular disease has a complex pathology, ischemia plays an intricate part in both the development and manifestation of cardiovascular disease. Ischemia induces several forms of cellular stress, including nutrient deprivation and hypoxia, that have all been linked to increases in posttranscriptional modifications of RNA.^3^, ^4^, ^5^, ^6^, ^7^

Small nucleolar RNAs (snoRNAs) are a type of small noncoding RNA that mediate RNA modifications at the post-transcriptional level. There are two types of snoRNAs, C/D box and H/ACA box snoRNAs, named after their conserved sequence motifs. C/D box snoRNAs guide 2′-O-ribose methylation (2′Ome) of their target RNAs. The DLK1-DIO3 locus on the long arm of human chromosome 14 encodes a cluster of 41 C/D box snoRNAs (14q32; 12F1 in mice). We have demonstrated that this cluster of 14q32 C/D box snoRNAs is strongly associated with vascular remodeling and human cardiovascular disease.^8^, ^9^, ^10^ The association with cardiovascular disease is both independent of and stronger than the 14q32 long noncoding RNAs (lncRNAs) and the cluster of 14q32 microRNAs that lie adjacent to the snoRNA genes.^8^ Furthermore, plasma levels of 14q32 snoRNAs were associated with disease outcome in patients with peripheral arterial disease (PAD).^9^^,^^10^ However, all 14q32 C/D box snoRNAs, except for one, are orphan snoRNAs, meaning that they have no known RNA targets.

We recently demonstrated that one of the most abundantly expressed snoRNAs of the 14q32 cluster, human SNORD113-6, and its murine equivalent, AF357425, target mRNAs of the integrin signaling pathway, influencing both pre-mRNA processing and 2′Ome. The D’ antisense box of AF357425/SNORD113-6 is fully conserved between humans and mice. Fibroblast integrin signaling is important for cell-cell and cell-matrix interactions and acts in various forms of cardiovascular remodeling that can lead to cardiovascular disease.^11^ Indeed, knockdown of SNORD113-6 altered human arterial fibroblast function.^12^

C/D box snoRNAs associate with four conserved ribonucleoproteins NHP2L1, NOP56, NOP58, and Fibrillarin (FBL). FBL is the methyltransferase that catalyzes 2′Ome. C/D box snoRNAs have two antisense boxes, located directly upstream of the D and D’ boxes, which are not covered by ribonucleoproteins and are thus free to interact with target RNA sequences. C/D box snoRNAs hybridize to their target RNAs via Watson-Crick base-pairing. Once bound to the target RNA, the 5^th^ nucleotide upstream of the D or D’ box is positioned for 2′Ome.^13^, ^14^, ^15^ Many expressed C/D box snoRNAs, however, lack antisense elements of known rRNA 2′Ome sites and are considered orphan snoRNAs.^16^ Likely, these orphan C/D box snoRNAs target other types of RNA molecules than rRNA.^17^, ^18^, ^19^

Besides rRNAs, transfer (t)RNAs are the most heavily modified cellular RNAs.^20^ Their canonical function lies in protein translation, where they deliver amino acids to the translating peptide chain. However, recent reports show that tRNAs can be processed into tRNA-derived fragments (tRFs), which can perform other, noncanonical, functions. tRFs can derive from different regions of their parental tRNA, located anywhere from the 5′ to 3′ end, and have variable sizes up to ∼50 nucleotides.^21^^,^^22^ Fragmentation of tRNAs can be induced under cellular stress, such as oxidative stress and hypoxia, which are important triggers of vascular remodeling processes.^23^, ^24^, ^25^ Among others, angiogenin (ANG) is a tRNA-processing endonuclease that is activated during cellular stress.^23^

Recent findings show that RNA modifications guided by snoRNAs can prevent tRNA cleavage and thereby regulate tRF formation.^21^^,^^26^, ^27^, ^28^, ^29^ For instance, SNORD97 induces 2′Ome on the wobble cytidine C34 of tRNA^Met^(CAT), which protects against ANG-induced cleavage.^26^ Whether AF357425/SNORD113-6 also guides 2′Ome on small RNA molecules like tRNAs, however, is still unknown.

Here, we aimed to determine whether AF357425/SNORD113-6 can target small RNAs in the vasculature, using primary murine fibroblasts (PMFs) and primary human umbilical arterial fibroblasts (HUAFs). Fibroblasts were chosen for their relevance in vascular remodeling^2^ and because the 14q32 snoRNAs are expressed most abundantly in fibroblasts.^16^ We used both murine and human fibroblasts to be able to look only at putative target RNAs that were conserved between the two species. We confirmed our key findings using an *ex vivo* model for ischemia in intact human arteries.^30^ We performed small RNA sequencing (sRNA-seq) on primary PMFs in which we either inhibited or overexpressed AF357425. We found that tRFs were the predominant group of small RNAs that changed in expression. Knockdown of AF357425 resulted in an apparent reduction of total tRFs but an enrichment of smaller sized tRFs (18–30 nucleotides). We focused on one of these tRNAs, tRNA leucine anti-codon TAA (tRNA^Leu^(TAA)), which has a predicted binding site for AF357425 in mice and SNORD113-6 in human. sRNA-seq data showed that its dominant tRF, tRF^Leu 47–64^, is formed just upstream of this site. Formation of tRF^Leu 47-–64^ was conserved in both PMFs and HUAFs and was investigated under oxidative, hypoxic, and starvation stress. We show that AF357425/SNORD113-6 indeed methylates this tRNA and protects against site-specific tRNA^Leu^(TAA) fragmentation.

## Results

### sRNA-seq in PMFs

**Figure 1:**
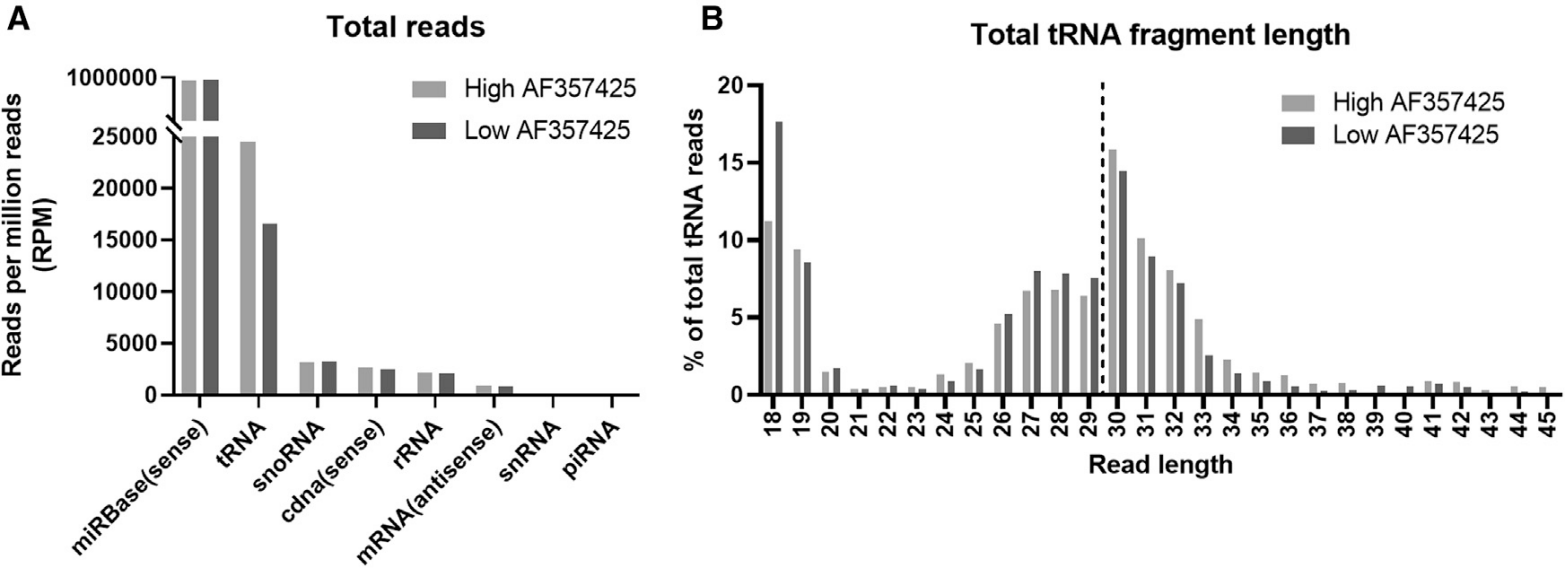
sRNA-seq (A) Total reads mapped to small RNAs, normalized to reads per million (RPM) and (B) total tRNA fragment length (in nucleotides), shown as a percentage (%) of total tRNA reads in high AF357425 (overexpressed) and low AF357425 (inhibited) primary murine fibroblasts. N.B., Piwi-interacting RNAs (piRNAs) and small nuclear RNAs (snRNAs) were detected, but the number of reads was too low to be visible in the graph at this y-axis scale.

In order to identify small RNA targets of AF357425/SNORD113-6, we performed sRNA-seq on PMFs in which we either inhibited or overexpressed AF357425. With this strategy, we aimed to obtain the largest possible difference in small RNA target expression. Gapmers were used to inhibit AF357425 expression (GM-AF25) and 3^rd^ generation antisense oligonucleotides (3GAs) to overexpress AF357425 (3GA-AF25). We showed previously that 3GAs directed against the 3′ end of AF357425 (3GA-AF25) induced snoRNA overexpression, likely through protection from degradation by endonucleases.^8^ Expression of AF357425 was increased (>5-fold) in PMFs treated with 3GA-AF compared with GM-AF25 (Figure S1). By far, most reads from the sRNA-seq in both samples were mapped to microRNAs. Read counts mapped to microRNAs, as well as those that mapped to the much lower expressed snoRNA and rRNA genes, were similar between AF25-high and AF25-low cells. However, reads that mapped to tRNA genes, which are all tRFs of <45 nucleotides in length, appeared to be reduced in number in AF25-low cells (Figure 1A; Tables S2 and S3). Where the longer tRFs (30–45 nucleotides) appeared enriched in AF25-high cells, smaller-sized tRFs (18–29 nucleotides) were enriched in AF25-low cells, particularly the 18-nucleotide tRFs (Figure 1B). These data indicate that AF357425 may protect against tRNA fragmentation.

### Fragmentation of tRNA^Leu^(TAA)

**Figure 2:**
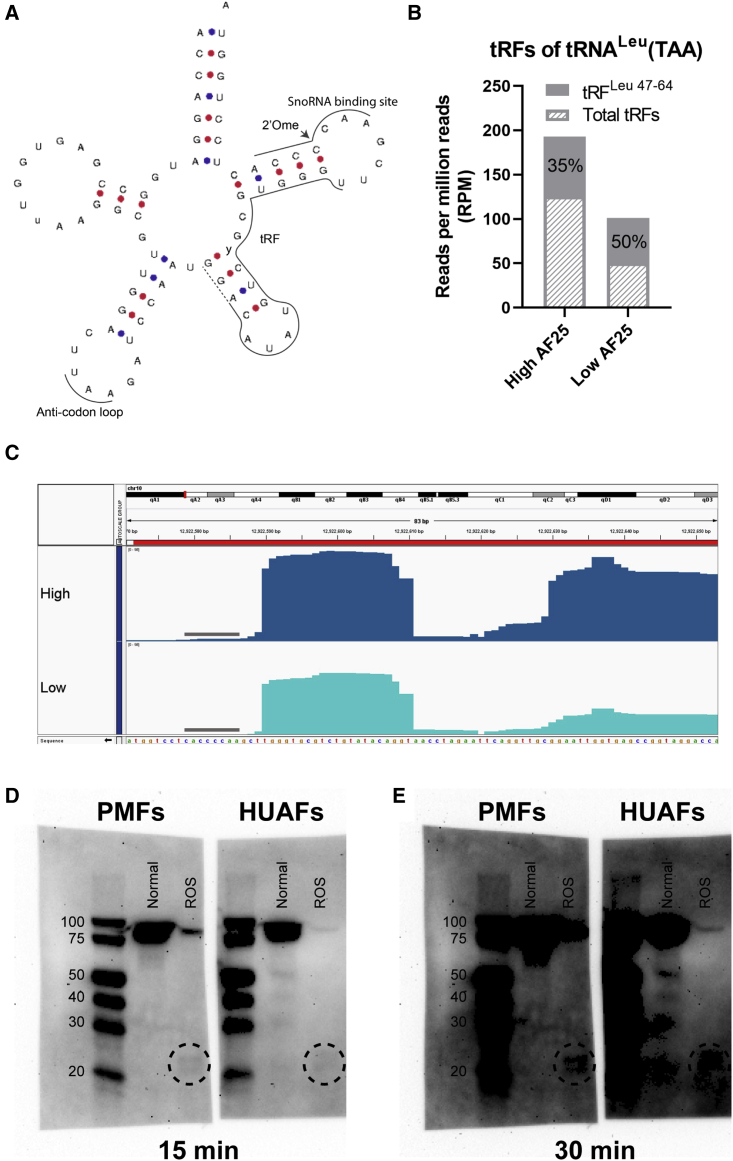
Fragmentation of tRNA^Leu^(TAA) (A) Schematic overview of the mature tRNA^Leu^(TAA) sequence in mouse and human. The AF357425/SNORD113-6 predicted binding site (snoRNA binding site), tRNA fragment generated upstream of the binding site, tRF^Leu 47–64^, and the anti-codon loop are indicated with a line. The dashed line indicates length extension of tRF^Leu 47–64^, representing 3 different sizes of generated tRFs. Arrow points at the predicted 2′O-methylation (2′Ome) site. γ represents U in mice and C in human. Figure adapted from GtRNAdb.^31^ (B) The fragment of interest, tRF^Leu 47–64^, shown as a percentage of total tRFs generated from tRNA^Leu^(TAA). Data are normalized to RPM. (C) Coverage plot of tRNA^Leu^(TAA) in primary murine fibroblasts with either high or low AF357425 expression. The predicted AF357425 binding site is indicated with a line. Sequence is shown from 3′ to 5′. (D and E) Northern blots exposed for (D) 15 and (E) 30 min using the ChemiDoc-IT imaging system. Primary murine fibroblasts (PMFs) and human umbilical arterial fibroblasts (HUAFs) were cultured in normoxic and reactive oxidative stress (ROS) conditions. A digoxigenin labeled probe was used to visualize tRFs. A circle indicates tRF^Leu 47–64^.

In order to investigate the mechanisms through which AF357425/SNORD113-6 may influence tRNA fragmentation, we focused on a single tRNA. Among others, tRNA^Leu^(TAA) had a predicted D’ box antisense sequence for AF357425. This site was conserved in human tRNA^Leu^(TAA) for the D’ box antisense sequence of SNORD113-6. Except for the outer 2 nucleotides, a perfect reverse complementary sequence for the middle 7 nucleotides is present in both mouse and human tRNA^Leu^(TAA) (AACCCCA; Figure 2B). A tRF cleaved just upstream of this predicted 2′Ome site (18–20 nucleotides; Figure 2A), tRF^Leu 47–64^, was abundantly expressed in both AF25-high and AF25-low cells (Figure 2B). However, the total tRFs generated from tRNA^Leu^(TAA) were decreased in AF25-low cells. In contrast, tRF^Leu 47–64^ was more abundant relative to the total tRFs in AF25-low (50%) compared with AF25-high cells (35%; Figures 2B and 2C). We confirmed expression of tRF^Leu 47–64^ by northern blot in both PMFs and in HUAFs. Expression of tRF^Leu 47–64^ appeared enhanced under oxidative stress (Figures 2D and 2E).

### Validation of tRNA^Leu^(TAA) 2′Ome

**Figure 3:**
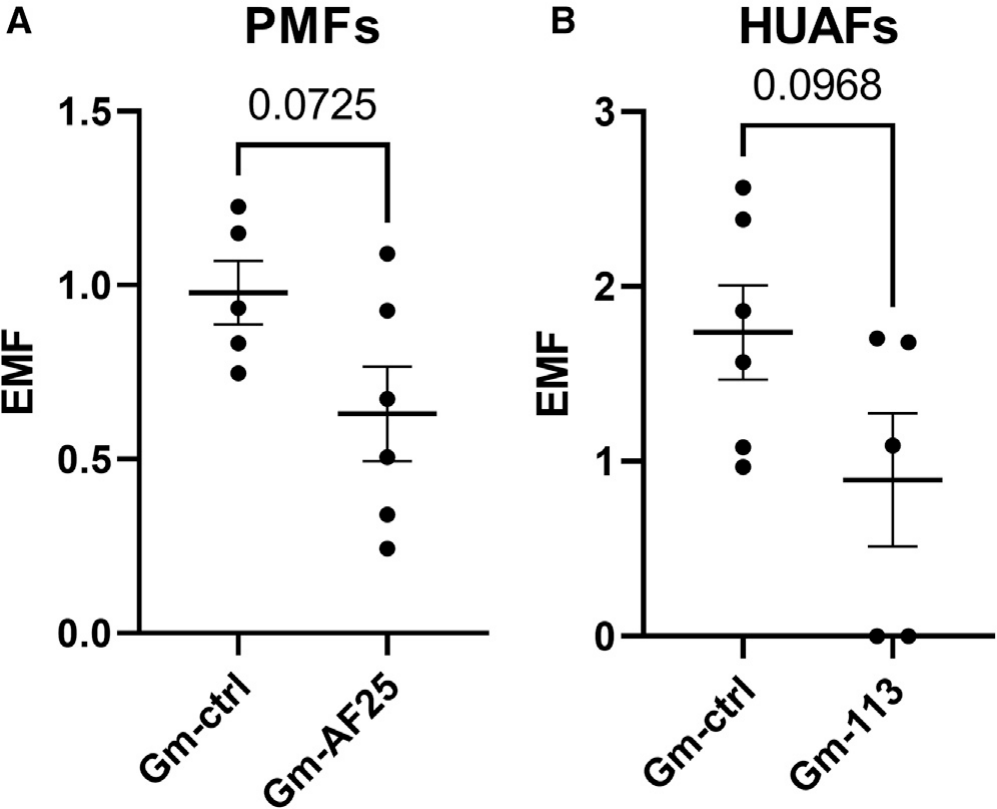
2′Ome at predicted AF357425/SNORD113-6 binding sites on tRNA^Leu^(TAA) For detection of 2′O-methyated nucleotides and to calculate the estimated methylated fraction (EMF), reversed transcription at low dNTP concentration followed by quantitative PCR (RTL-Q) was performed. Site-specific reversed primers were used exactly on and 1 nucleotide downstream of the predicted 2′Ome site to accurately determine the exact location of the 2′Ome site. (A) PMFs or (B) HUAFs were transfected with gapmers against AF357425 (GM-AF25)/SNORD113-6 (GM-113) or a gapmer control (GM-ctrl) for 24 h. Data represent a minimum of three independent experiments. n is represented by the individual dots. Data are represented as mean ± SEM. A two-tailed unpaired t test was performed to compare GM-AF25/113 with GM-ctrl.

Next, we performed reverse transcription at low dNTP concentration followed by quantitative PCR (RTL-Q) to calculate the estimated methylated fraction (EMF), using site-specific primers for detection of 2′Ome. We confirmed 2′Ome of the mature full-length tRNA^Leu^(TAA), located on the 5^th^ nucleotide upstream of the D’ antisense box, in both PMFs and HUAFs. Inhibition of AF357425/SNORD113-6 partly reduced 2′Ome at this site compared with a gapmer control (GM-ctrl; p for trend = 0.0725 in PMFs and 0.0968 in HUAFs; Figure 3). 2′Ome also appeared present on the precursor-(pre)tRNA^Leu^(TAA) in HUAFs, but we could not confirm snoRNA-induced regulation of 2′Ome in the pre-tRNA^Leu^(TAA) due to high Ct values above the detection threshold (>45 Ct; Figure S2). The validation of 2′Ome in both mouse and human cells suggests that both are evolutionarily conserved features of tRNA^Leu^(TAA).

### Fragmentation of tRNA^Leu^(TAA) under cellular stress

**Figure 4:**
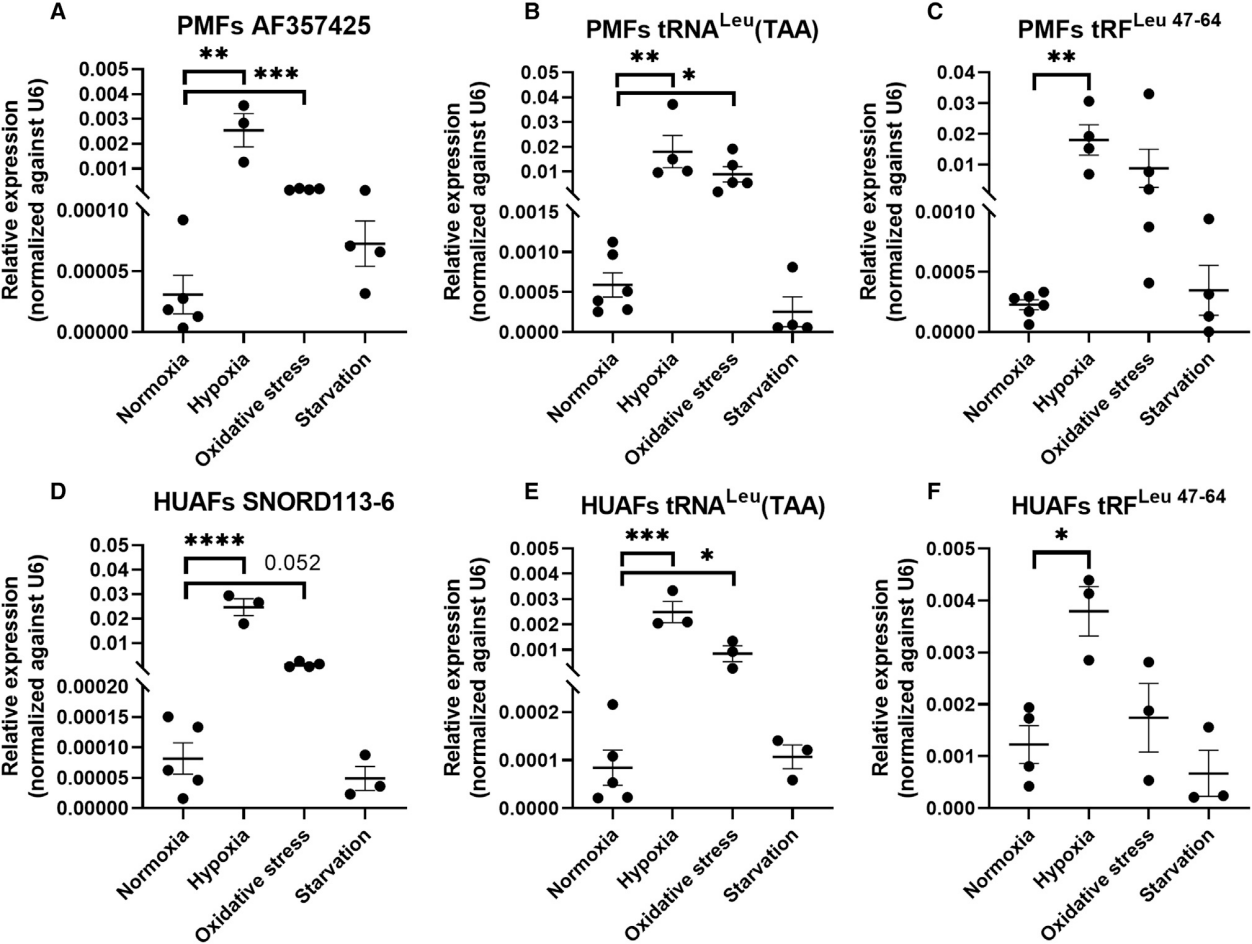
Relative snoRNA, tRNA^Leu^(TAA), and tRF^Leu 47–64^ expression during cellular stress Relative expression levels in (A–C) PMFs and (D–F) HUAFs cultured in normoxic, hypoxic, oxidative stress, and starvation conditions. Expression levels are normalized to U6. Data represent a minimum of three independent experiments. n is represented by the individual dots. Data are represented as mean ± SEM. A two-tailed unpaired t test was performed to compare treatment with the control. ∗p < 0.05, ∗∗p < 0.01, ∗∗∗p < 0.005, compared with normoxia.

As fragmentation of tRNAs can be induced during cellular stress,^23^, ^24^, ^25^ we cultured PMFs and HUAFs under different cellular stress conditions and measured expression levels of AF357425/SNORD113-6, mature tRNA^Leu^(TAA), and tRF^Leu 47–64^ by qPCR. Endogenous expression of AF357425/SNORD113-6 and mature tRNA^Leu^(TAA) showed similar expression patterns in PMFs and HUAFs, with increased expression under both hypoxia and oxidative stress, compared with the normal culture condition control. Serum starvation, on the other hand, did not induce changes in either AF357425/SNORD113-6 or tRNA^Leu^(TAA) expression compared with normal culture conditions. Expression of tRF^Leu 47–64^ was only increased significantly under hypoxia in both PMFs and HUAFs but appeared slightly elevated under oxidative stress as well (Figure 4).

**Figure 5:**
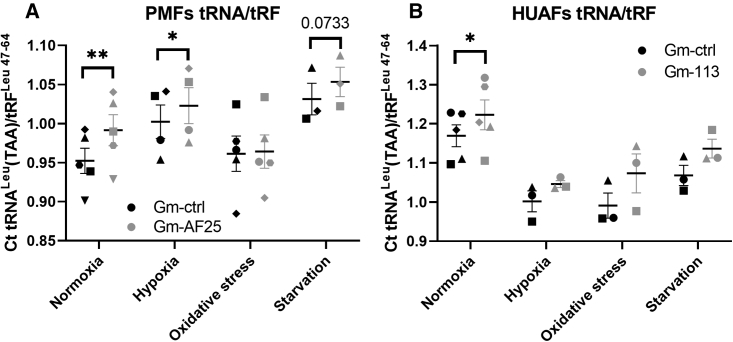
Ratio mature tRNA^Leu^(TAA)/tRF^Leu 47–64^ in primary cells exposed to different cell stress stimuli (A) PMFs and (B) HUAFs transfected with GM-AF25/113 or GM-ctrl and cultured in normoxic, hypoxic, oxidative stress, or starvation conditions. Expression levels were measured by qPCR. Ratios were calculated by dividing absolute Ct values of the mature tRNA by Ct values of the tRF. Data represent a minimum of three independent experiments. n is represented by the individual dots. Data are represented as mean ± SEM. A two-tailed paired t test was performed to compare single treatment with the control, within each experiment. ∗p < 0.05, ∗∗p < 0.01, compared with GM-ctrl.

Subsequently, PMFs and HUAFs were transfected with either GM-AF25/113 or a GM-ctrl and were cultured under the different cell stress conditions. The absolute Ct value of mature tRNA^Leu^(TAA) was divided by the Ct value of tRF^Leu 47–64^ in order to quantify the expression of tRF^Leu 47–64^ relative to mature tRNA^Leu^(TAA), which it was generated from. The ratio was increased under AF357425/SNORD113-6 inhibition under control conditions in both PMFs and HUAFs (Figure 5). In PMFs, the ratio was also increased under hypoxia and showed a trend toward an increased ratio under serum starvation (Figure 5A). This increased ratio demonstrates that more tRF^Leu 47–64^ is formed relative to its mature tRNA, when AF357425/SNORD113-6 is inhibited. When we quantified expression of ANG, we did not observe a difference between GM-AF25/113 and GM-ctrl (Figure S3). However, ANG did increase under cellular stress, similar to the snoRNA, both in PMFs and HUAFs. We can neither confirm nor exclude that ANG is responsible for cleavage of tRNA^Leu^(TAA), but we can conclude that ANG is not influenced by the snoRNA directly and that changes in fragmentation are likely caused by changes in snoRNA-guided 2′Ome of the tRNA.

### snoRNA, tRNA, and tRF expression in human arteries

In many vascular pathologies, adventitial fibroblasts are activated and become myofibroblasts.^2^ We therefore examined whether inhibition of SNORD113-6 affects this phenotype transition (Figure S4). There was a trend toward reduced collagen type 1 alpha 1 chain (COL1A1) expression in GM-113-treated fibroblasts (p = 0.07); however, protein expression was affected by snoRNA inhibition. Although α-smooth muscle actin (αSMA) was expressed, as is common for fibroblasts in culture, we did not observe differences between GM-113- and GM-ctrl-treated cells.

**Figure 6:**
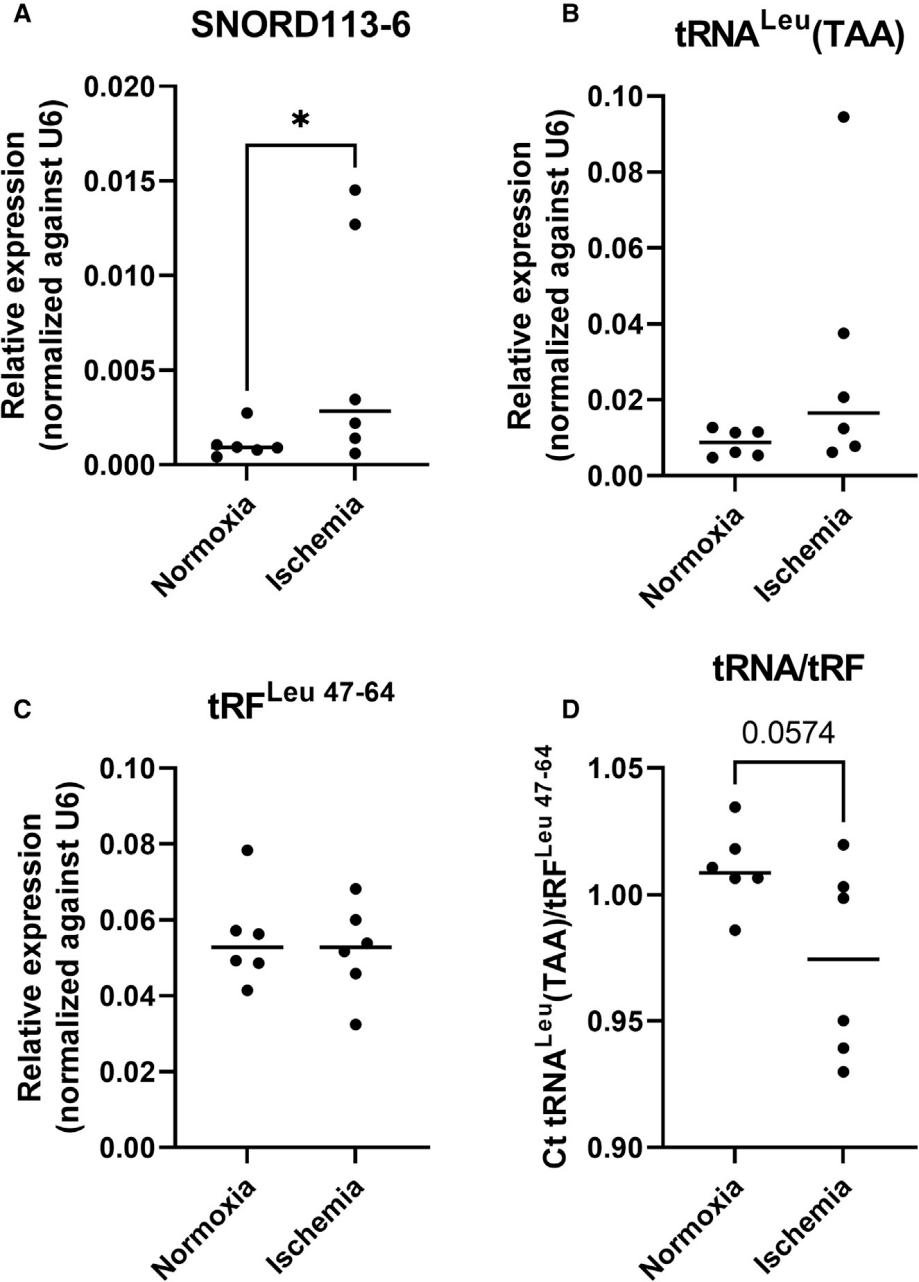
Relative snoRNA, tRNA^Leu^(TAA), and tRF^Leu 47–64^ expression in human inner mammary arteries cultured *ex vivo* under ischemic conditions Relative expression levels of SNORD113-6 (A), tRNA Leu(TAA) (B), and tRFLeu 47–64 (C), as well as ratios calculated by dividing absolute Ct values of the mature tRNA by Ct values of the tRF (D). n is represented by the individual dots. Data are represented as mean ± SEM. A two-tailed ratio paired t test was performed to compare expression between normoxic and ischemic artery samples. ∗p < 0.05.

To examine whether tRNA^Leu^(TAA) fragmentation increases under stress conditions in human vascular tissues, we used internal mammary arteries (IMAs), which were harvested during elective coronary bypass surgery on patients with coronary artery disease. After culturing the vessels *ex vivo* under control or ischemic (= hypoxia + starvation) conditions for 24 h, expressions of SNORD113-6, tRNA^Leu^(TAA), and tRF^Leu 47–64^ were measured. Consistent with our *in vitro* models, expression of SNORD113-6 was significantly upregulated (p = 0.0432) under ischemic conditions (Figure 6A). tRNA^Leu^(TAA) also appeared somewhat upregulated, but not significantly (Figure 6B). tRF^Leu 47–64^ expression, however, was not altered (Figure 6C). When we calculated the Ct tRNA^Leu^(TAA)/tRF^Leu 47–64^, there was a trend toward a decrease in the ratio, indicating that less tRNA^Leu^(TAA) was cleaved into tRF^Leu 47–64^ (Figure 6D).

### Effects of 2′Ome on tRNA stability

**Figure 7:**
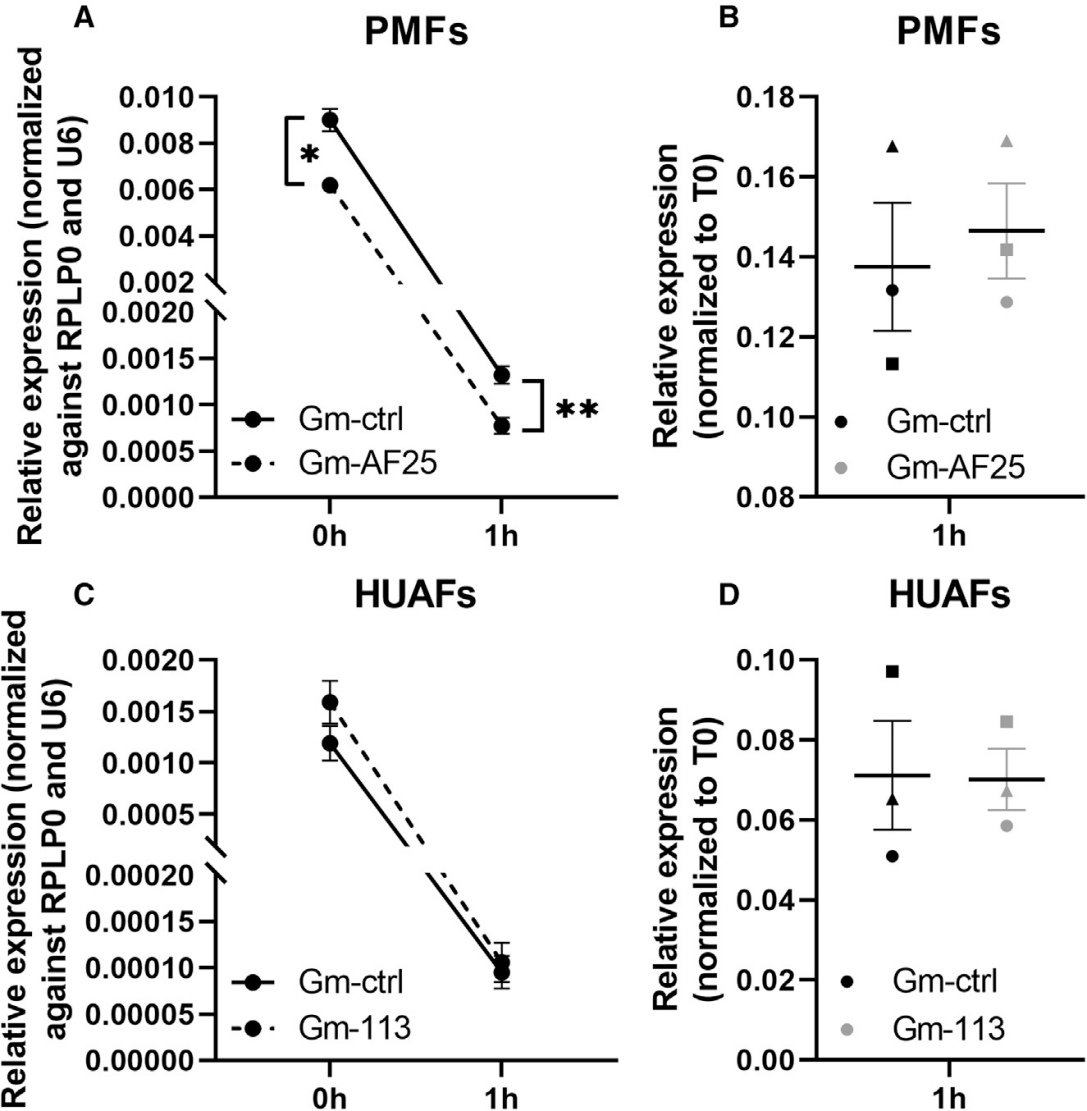
Stability of tRNA^Leu^(TAA) in primary cells (A and B) PMFs and (C and D) HUAFs were transfected with GM-AF25/GM-113 or a GM-ctrl for 24 h. After 24 h, cells were treated for 1 h with actinomycin D to inhibit novel RNA transcription. Mature tRNA levels were measured 0 and 1 h after addition of actinomycin D. (A and C) Expression levels were normalized to RPLP0 and U6. n is 3. (B and D) Expression levels at 1 h were normalized to 0 h (T0). n is represented by the individual dots. Data are represented as mean ± SEM. ∗p < 0.05, ∗∗p < 0.01, compared with GM-ctrl.

Ribonucleotide modifications in the structural core of the tRNA may stabilize the tRNA and reduce tRNA degradation rates.^32^ After transfection with either GM-AF25/113 or GM-ctrl, cells were treated with a high concentration of actinomycin D (5 μg/μL) for 1 h to inhibit novel tRNA transcription. In both PMFs and HUAFs, mature tRNA^Leu^(TAA) was rapidly degraded, but no differences were observed between GM-AF25/113 and GM-ctrl (Figures 7A and 7C). In PMFs, the relative expression of mature tRNA^Leu^(TAA) was lower to begin with in AF25-low cells and remained lower after 1 h, compared with GM-ctrl (Figure 7A). To rule out differences in degradation rates of housekeeping genes used (RPLP0 and U6) between the two groups, we also normalized the expression levels at 1 to 0 h (T0; Figures 7B and 7D). Indeed, no differences in degradation rates between GM-AF25/113 or GM-ctrl were observed, indicating that a reduction of this single 2′Ome modification did not affect overall mature tRNA^Leu^(TAA) stability but only the site-specific fragmentation.

## Discussion

Here, we aimed to further elucidate the role of the vasoactive 14q32 snoRNA AF357425/SNORD113-6 in vascular remodeling. We investigated whether AF357425/SNORD113-6 could also target small RNAs besides the previously identified integrin signaling mRNA targets.^33^ We found that tRNAs are the predominant small RNA target of AF357425 in primary fibroblasts. Inhibition of AF357425/SNORD113-6 led to an overall decrease in tRFs, and, compared with overexpression of the snoRNA, less larger (>30 nucleotides) and more smaller (<30 nucleotides) tRFs were formed. In order to investigate the underlying mechanisms of action, we focused on tRNA^Leu^(TAA), which has a conserved binding site for the D’ box seed sequence of AF357425/SNORD113-6. We showed that tRNA^Leu^(TAA) is a 2′Ome target of AF357425/SNORD113-6 and that snoRNA inhibition led to an apparent reduction of 2′Ome at this site, both in murine and in human primary fibroblasts. Endogenous expression of AF357425/SNORD113-6 and mature tRNA^Leu^(TAA) both increased under hypoxia and oxidative stress. Endogenous tRF^Leu 47–64^ expression was also elevated under hypoxia *in vitro*. In intact human arteries however, ischemia-induced upregulation of SNORD113-6 appeared to reduce tRNA^Leu^(TAA) fragmentation into tRF^Leu 47–64^. Knockdown of AF357425/SNORD113-6 resulted in an increased ratio of tRF^Leu 47–64^ relative to its mature tRNA^Leu^(TAA). 2′Ome by AF357425/SNORD113-6 was not important for the overall stability of the tRNA, and therefore, we conclude that it acts via protecting against site-specific fragmentation of tRNA^Leu^(TAA) into tRF^Leu 47–64^.

Regarding the biological role of tRF^Leu 47–64^ in vascular remodeling and cardiovascular disease, much remains unclear. Just like we show here, several other recent studies have shown that fragmentation of tRNAs increases under cellular stress.^23^, ^24^, ^25^^,^^34^ Similar cellular stress conditions also trigger vascular remodeling. This implicates that the formation of tRFs play a regulatory role in vascular remodeling. Indeed, oxygen-glucose deprivation, which is an *in vitro* model for ischemic reperfusion injury, induced tRNA cleavage in neuronal cells.^25^ Also *in vivo*, in models for acute ischemic stroke and PAD, the formation of tRFs was strongly increased.^24^ Similarly, we have previously shown that the 14q32 snoRNAs are regulated under ischemic conditions in patients with PAD and during vascular remodeling.^8^ In the current study, we show that expression of AF357425/SNORD113-6 indeed increased during hypoxia and oxidative stress in cells, as well as under ischemia in whole human inner mammary arteries cultured *ex vivo*. In whole artery tissue, ischemia appear to reduce the cleavage of tRNA^Leu^(TAA) into tRF^Leu 47–64^ when looking at their Ct ratio. In cells, we did not observe this decrease; however, exposing cells to cellular stress did not result in an additional increase of tRF^Leu 47–64^ to mature tRNA^Leu^(TAA) ratio in AF357425/SNORD113-6 knockdown cells, whereas expression of mature tRNA^Leu^(TAA) and ANG both increased under both hypoxia and oxidative stress.

Even though our findings implicate a role for site-specific fragmentation of tRNAs in vascular remodeling, the question remains what the molecular and biological function of the formed tRNA fragment tRF^Leu 47–64^ could be. Assumingly, tRF^Leu 47–64^ has an important role in vascular biology, as tRF^Leu 47–64^ is also generated under physiological conditions and not exclusively during cellular stress. Our group, as well as others, have shown that tRFs have potential as circulating biomarkers in, for example, acute stroke.^35^, ^36^, ^37^ It has also been demonstrated that tRFs can perform all sorts of regulatory functions, including regulation of protein translation, microRNA-like functions by base-pairing with mRNAs, and interaction with RNA-binding proteins.^38^^,^^39^ Furthermore, tRFs have been shown to be functionally active in modulating cardiac and skeletal muscle function and endothelial function but also in inhibition of angiogenesis.^24^^,^^40^ Which exact regulatory function(s) tRF^Leu 47–64^ may have and how it impacts vascular function, as well as vascular remodeling, remains to be determined.

Regarding the molecular role of snoRNA-induced 2′Ome of tRNA^Leu^(TAA), the same post-transcriptional modifications on tRNAs have been shown to both protect from and promote fragmentation.^27^^,^^28^ Our data suggest that AF357425/SNORD113-6 2′Ome protects the tRNA from cleavage into small fragments (∼18 nucleotides in length) rather than promoting it. However, we found more tRFs in total, including all tRFs formed of tRNA^Leu^(TAA), and longer fragments (>30 nucleotides) in AF357425-high cells than in AF357425-low cells. Perhaps the presence or absence of modifications attracts different tRNA endonucleases, which produce different tRF species. The role of AF357425/SNORD113-6 2′Ome in cleavage of other tRNAs remains to be determined, but likely, its function is to prevent fragmentation of shorter tRFs (∼18 nucleotides in length).

Besides the stabilizing tertiary structure, little is known about other functions of post-transcriptional modifications in the structural core of tRNAs.^32^ AF357425/SNORD113-6 targets and guides 2′Ome in the T-arm, the structural core, of tRNA^Leu^(TAA). 2′Ome and the formation of tRF^Leu 47–64^ were found in both mice and humans, suggesting that both are evolutionarily conserved features of tRNA^Leu^(TAA). Degradation rates of tRNA^Leu^(TAA) were similar between GM-ctrl and GM-AF25/113 transfected cells, indicating that 2′Ome by AF357425/SNORD113-6 is not important for the overall tRNA^Leu^(TAA) stability. Of course, tRNAs are heavily modified, and reduction of a single 2′Ome modification may not have direct consequences for their stability. This could however pose a threat to the reliability of our measurements. Modifications on tRNAs may impede reverse transcription and limit detection of tRFs and tRNAs by qRT-PCR.^41^ However, 2′Ome by AF357425/SNORD113-6 is located toward the 3′ end of the tRNA. We designed qPCR primers for mature tRNA^Leu^(TAA) upstream of that site in order to limit confounding effects by the presence or absence of 2′Ome. Furthermore, expression of tRF^Leu 47–64^, which we initially found in the sRNA-seq, was confirmed by northern blot, and our qPCR results showed that tRF^Leu 47–64^ and mature tRNA^Leu^(TAA) were both abundantly expressed. We cannot control for effects of other modifications in our qPCRs; however, if reversed transcription was hampered by other modifications, these were likely similar between GM-AF25/113 and GM-ctrl.

Taken together, we show that AF357425/SNORD113-6 targets predominantly tRNAs, protecting the tRNA from cleavage into small fragments. When zooming in on one specific tRNA, tRNA^Leu^(TAA), we show that AF357425/SNORD113-6 induces 2′Ome of the mature tRNA, thereby protecting against site-specific tRNA fragmentation. The function of tRF^Leu 47–64^ in vascular remodeling and whether this tRF forms a potential future therapeutic target for treatment and/or prevention of cardiovascular disease, however, remain to be elucidated.

## Materials and methods

### Cell culture

Cells were cultured in a humidified incubator at 37°C under 5% CO_2_. Cells were passaged at 70%–90% confluency and used up to passage 6. DMEM, supplemented with 10% heat-inactivated fetal calf serum (FCSi) and 1% Pen/Strep, was used as culture media and was refreshed every 2–3 days.

### PMF isolation

Ear tissues from C57BL/6-J mice, about 3 weeks of age, were clipped into smaller pieces and embedded in 0.2% gelatine in 6-well plates. DMEM supplemented with 20% FCSi and 1% nonessential amino acids (NEAAs; Thermo Fisher Scientific, Waltham, MA, USA, cat. no. 11140050) was added to the embedded ear clippings. After 7 days, skin fibroblasts were grown out of the tissues onto the bottom of the culture plates. PMFs were expanded in culture media up to passage 3. PMFs were then used for further analysis or frozen down and stored in liquid nitrogen for later use.

### Primary HUAF isolation

Umbilical cords from full-term pregnancies were collected, stored in sterile PBS at 4°C, and, within 7 days, used for HUAF isolation. The two arteries were isolated from the umbilical cord. Endothelial cells were removed by gently rolling the artery over a blunted needle. After that, the tunica adventitia and tunica media were separated using surgical tools. The tunica adventitia was incubated overnight in culture media supplemented with 10% heat inactivated human serum (PAA, Pasching, Austria) and 1% NEAA. The next day, the tunica adventitia was treated with 2 mg/mL collagenase type II solution (Worthington, OH, USA, cat. no. NC9693955) at 37°C. The resulting cell suspension was filtered over a 70 μm cell strainer and centrifuged at 400 × *g* for 10 min. Cells were plated in 6-well plates, and media were refreshed after 90 min to remove slow adhering nonfibroblast cells. HUAFs were expanded up to passage 3 and used for further analysis or frozen down and stored in liquid nitrogen for later use.

### Cellular stress conditions

Oxidative stress in both PMFs and HUAFs was induced by adding 10 μM reactive oxygen species (ROS) mimic tert-butyl hydroperoxide (tBHT; Luperox, 458139, Sigma Aldrich, St. Louis, MO, USA) to the culture media for 24 h. To serum starve the cells, DMEM with 1% PenStrep, supplemented with 1% FCSi for PMFs or 3% FCSi for HUAFs, was added to the cells. Different incubation times were tested for serum starvation (Figure S5). An incubation time of 24 h was determined as the most optimal time point. To induce hypoxia, cells in normal culture media were kept in a humidified incubator at 37°C under 1% O_2_ for 24 h. Absolute Ct values of AF357425 and U6 expression are shown in Figure S6.

### RNA isolation and qRT-PCR

RNA was isolated by standard TRIzol (Thermo Fisher Scientific, cat. no. 15596026) chloroform extractions. RNA concentration and purity were measured using Nanodrop (Nanodrop Technologies, Wilmington, DE, USA) or the Bioanalyzer (2100 Bioanalyzer Instrument, Agilent, Santa Clara, CA, USA).

Reversed transcription of total RNA was performed with the high-capacity RNA-to-cDNA reverse transcription kit (Applied Biosystems, Thermo Fisher Scientific, cat. no. 4388950). Quantitect SybrGreen reagents (Qiagen Benelux, Venlo, the Netherlands, cat. no. 204145) were used for quantifications. Custom designed TaqMan small RNA assays (Thermo Fisher Scientific, cat. no. 4398987) were used for reversed transcription and quantifications of tRFs. Expression levels were normalized to U6 using the 2^−ΔCt^ method. All primers used are provided in Table S1.

### 3GAs and gapmers

3GAs were kindly provided by Idera Pharmaceuticals (Cambridge, MA, USA). 3GAs directed to AF357425 consisted of two identical strands of DNA nucleotides with a full phosphorothioate backbone, connected by a 5′ phosphorothioate linker. GMs were custom designed against AF357425 (GM-AF25) or SNORD113-6 (GM-113; Sigma Aldrich). GMs were made up out of five 2′Ome RNA nucleotides, 10 DNA nucleotides, and five more 2′Ome RNA nucleotides with full phosphorothioate backbone. Sequences of 3GAs and GMs are provided in Table S1.

### Transfection with 3GAs and gapmers

Prior to transfections, G1 cell-cycle arrest was induced by treating cells with KN-93 (Sigma Aldrich, cat. no. K1385), an inhibitor of CaMK-II (the multifunctional Ca^2+^/CaM kinase). KN-93 was added to the culture media at a concentration of 10 μM for 48 h. After cell synchronization, cells were washed with PBS, and basal DMEM was added. Meanwhile, lipofectamine RNAiMAX Reagent (Thermo Fisher Scientific, cat. no. 13778030) was used to create micelles loaded with 3GAs or GMs against snoRNA AF357425 or SNORD113-6 for transfection. Micelles were added to the cells, and after 1 h of transfection, 10% FCSi was supplemented to the transfected cells. Two concentrations of GM transfection were tested for optimal snoRNA inhibition (Figure S7). The optimal inhibition, without visible cytotoxic effects, was at 500 nM. A timeline of AF357425 and SNORD113-6 inhibition showed efficient snoRNA knockdown at 24 h. Therefore, for all experiments a concentration of GMs (500 nM) or 3GAs (200 nM; as established in a previous study^8^) and a total transfection time of 24 h were used. After 24 h of transfection, cells were washed with PBS and used for further experiments or analyses.

### RNA-seq and analysis

RNA was isolated from PMFs transfected with 3GAs or GMs against AF357425. Absolute Ct values of AF357425 and U6 expression are shown in Table S4 and Figure S6. Isolated RNA was shipped to BGI for DNBseq sRNA-seq (GEO: GSE190537). Generated sRNA-seq files in FASTQ format are processed using the sRNAbench tool.^42^ Bowtie aligner was used to align reads to various reference genome and databases, such as GRCm38, mirbase small database, and RNAcentral. The expression of multiple classes of small RNA are quantified in the single assignment-based approach where reads mapping to multiple loci are assigned to the locus that has the highest expression. Reads per million (RPM) normalized counts are further generated that are used for downstream analysis.

### Northern blotting

Total RNA samples were diluted in Novex Tris-borate-EDTA (TBE)-urea sample buffer (Thermo Fisher Scientific, cat. no. LC6876), denatured at 95°C for 5 min, and put on ice. 15% Mini-PROTEAN TBE-urea gels (BioRad, cat. no. 4566053) in TBE buffer were pre-run at 200 V for 20 min. After that, RNA samples and a digoxigenin (DIG)-labeled Blue Color Marker for small RNA (DynaMarker, BioDynamics, cat. no. DM270-125uL) were loaded on the gel. Gels were electrophoresed at 200 V for ∼1 h. Next, RNA was transferred from the gel to a Hybond N+ membrane (GE Healthcare, cat. no. RPN203B) at 200 mA for 1 h. A Mini Trans-Blot Electrophoretic Transfer Cell (BioRad) system with an ice element and stirrer were used for RNA transfer. Next, RNA was crosslinked to the membrane with freshly prepared 1-ethyl-3-(3-dimethylaminopropyl) carbodiimide (EDC; Sigma, cat. no. E1769) 1-methylimidazole (Sigma, cat. no. 336092) crosslinking solution (pH 8) for 1 h at 60°C. Membranes were pre-hybridized in ULTRAhyb Oligo Hybridization Buffer (Invitrogen, cat. no. AM8663) at 37°C for 30 min while gently shaking. Dual DIG-labelled DNA probes (designed and ordered at Integrated DNA Technologies, Coralville, IA, USA) were denatured at 95°C for 1 min, added to the hybridization buffer (final concentration 5 nM), and left overnight at 37°C. The next day, membranes were washed with low stringency wash buffer (2× SSC, 0.1% SDS) and high stringency wash buffer (0.1× SSC, 0.1% SDS) at 37°C and then washed with 2× SSC buffer at room temperature. Then, membranes were washed and blocked with the DIG Wash and Block Buffer Set (Roche, cat. no. 11585762001) according to the manufacture’s protocol. After blocking for 3 h at room temperature while shaking, anti-DIG-AP, Fab fragments (Roche, cat. no. 11093274910) in blocking buffer (1:15.000) were added to the membranes. CDP-star Development Reagent (Roche, cat. no. CDP-RO) was added to the membranes, and images were acquired using ChemiDoc-IT imaging system. Dual DIG-labelled DNA probes are listed in Table S1.

### Detection of 2′Ome

For detection of 2′Ome nucleotides, we used an adaptation of the RTL-Q method that was described by Dong et al.^43^ To accurately determine the exact location of the 2′Ome site on mature tRNA, a reversed primer downstream of the 2′Ome site (R_D_) and a reversed primer on the 2′Ome site (R_U_) were designed to the +1 and 0 nt downstream of the predicted 2′Ome nucleotide, respectively. The RT reaction was performed in two consecutive steps. First, a mixture of 20 ng RNA and 10 μM R_D_ or R_U_ primers was denatured at 70°C for 5 min and incubated at 42°C for 10 min as an initial annealing step. Then, a high (200 μM) or low (0.5 μM) concentration of dNTPs (Promega, cat. no. U1511), 200 U M-MLV reverse transcriptase (Promega, cat. no. M1705), and 20 U recombinant RNasin ribonuclease inhibitor (Promega, cat. no. N2515) was added to the RT reaction. The RT reaction was incubated at 42°C for 90 min, followed by incubation at 75°C for 15 min. When a 2′Ome site is present, the extension of the R_D_ primer pauses at this site when low dNTP concentrations are used, whereas the R_U_ primer does not. Primer extensions are not affected by 2′Ome sites when performing RT at high dNTP concentrations. The differences in RT products were quantified by SYBR green-based qPCR. The EMF was calculated using the following formula.

EMF = (Ct Low dNTP R_D_ – Ct High dNTP R_D_) – (Ct Low dNTP R_U_ – Ct High dNTP R_U_)

EMF >0 means that methylation is present. EMF ≤ 0 means that no methylation is present.

The sequence of the human precursor tRNA (pre-tRNA) was obtained from publicly available RNA-seq data performed by Gogakos et al.^44^ RT primers were designed around the predicted 2′Ome site. One reverse primer was designed upstream of the possible 2′Ome site (R_U_) and one downstream of the 2′Ome site (R_D_). One forward primer (FW) was used for both R_U_ and R_D_. The same RTL-Q conditions were used as for mature tRNA. All primer sequences are provided in Table S1.

### Fibroblast-to-myofibroblast transition

HUAFs were cultured on coverslips and transfected with GM-ctrl or GM-113-6 for 24 h. For collagen staining, cells were fixed in Zinc Formal-Fixx (Thermo Fisher Scientific, cat. no. 6764255) for 30 min and washed with PBS. Collagen was visualized with a picrosirius red staining.

For αSMA staining, cells were fixed in 4% PFA for 15 min and washed in PBS. An antibody against αSMA, 1A4 (Dako M0851, 1:1000), and a secondary antibody Alexa Fluor 555 DαMouse (Invitrogen A31570, 1:1000) were used to visualize αSMA. Hoechst (34580, 1:1,000) was used to stain the nuclei.

Fiji was used to perform immunohistochemistry and immunofluorescence analysis. The area was divided by the total amount of nuclei. The integrated density, which is the sum of values of the pixels, was calculated and divided by the total amount of nuclei (intensity).

Primer sequences for COL1A1 and αSMA (smooth muscle α-2 actin [ACTA2]) are provided in Table S1.

### Collection of surplus human artery samples

Human artery samples were collected at the Leiden University Medical Center. Collection, storage, and processing of the samples were performed in compliance with the Medical Treatment Contracts Act (WGBO, 1995) and the Code of Conduct for Health Research using Body Material (Good Practice Code, Dutch Federation of Biomedical Scientific Societies, 2002) and the Dutch Personal Data Protection Act (WBP, 2001).

Human IMAs were harvested during elective coronary bypass surgery from seven patients with coronary artery disease. Only surplus tissue was collected. These samples were anonymized, and no data were recorded that could potentially trace back to an individual’s identity. Vessels were left to rest overnight in culture medium (DMEM Glutamax with 10% heat FCSi and 100 U penicillin and 100 μg streptomycin per mL) at 37°C and 20% oxygen and subsequently cultured for 24 h, either at control conditions (20% oxygen and culture medium) or at hypoxia + starvation conditions (1% oxygen and FCS reduced to 0.5%).

Samples were snap frozen and stored at −80°C. Frozen tissues were crushed in liquid nitrogen, and total RNA was isolated from tissue powder by standard TRIzol-chloroform extraction as described above.

### Degradation assay

HUAFs and PMFs were treated with KN-93 for 48 h and then transfected with GM-113 or GM-AF25, respectively, or a negative GM-ctrl, as described above. After 24 h of transfection, cells were treated with 5 μg/μL actinomycin D (Sigma Aldrich, cat. no. A9415) to inhibit novel RNA transcription for 1 h. The decline of mature tRNA and the tRF levels over time were quantified by qPCR. Relative AF357425 and SNORD113-6 expression before and after 1 h actinomycin D treatment is shown Figure S8.

### Statistical analyses

Results are expressed as mean ± standard error of the mean (SEM). An unpaired t test was performed to compare single treatment with the control. As knockdown efficiency varied per experiment, for these experiments a paired t test was performed to compare each treatment with its own control, within each individual experiment. Graphpad (v.9.0.1) was used to perform all statistical analysis. p <0.05 was considered significant, and p < 0.1 was considered a trend.

## Data Availability

The small RNA-seq data sets of PMFs with overexpression (high) and inhibition (low) of AF357425 were deposited in the Gene Expression Omnibus database under GEO: GSE190537.

## Supplemental information

Document S1. Tables S1–S4 and Figures S1–S8

Document S2. Article plus supplemental information
