# Supplementary material for: C/D box snoRNA SNORD113-6 guides 2′-O-methylation and protects against site-specific fragmentation of tRNALeu(TAA) in vascular remodeling
Source: Mol Ther Nucleic Acids. 2022 Sep 17;30:162–72. doi: 10.1016/j.omtn.2022.09.011 (PMC9547152; doi:10.1016/j.omtn.2022.09.011)
Supplement: Document S1. Tables S1–S4 and Figures S1–S8 [file mmc1.pdf]

OMTN, Volume *30*

Supplemental information

C/D box snoRNA SNORD113-6 guides 2′-O-methylation and protects against site-specific fragmentation of tRNA^{Leu}(TAA) in vascular remodeling

Eva van Ingen, Pleun A.M. Engbers, Tamar Woudenberg, M. Leontien van der Bent, Hailiang Mei, Johann Wojta, Paul H.A. Quax, and A. Yaël Nossent

Supplemental Tables

Table S1. Oligonucleotide and primer sequences. 3rd Generation Antisense (3GA) and gapmers (GM). $[nnn]_D$ = DNA-nucleotides; $[nnn]_{2'OmeR}$ = 2'Ome-RNA nucleotides; X = phosphorothioate linker.

**Oligonucleotide**	
3GA-AF357425	3'-$[GGGTTTAATCACTGTCCTC]_D$-X-$[CTCCTGTCACTAATTTGGG]_D$-3'
GM-AF357425-D'	3'-$[GUCAG]_{2'OmeR}[AAACCCCATG]_D[CUCCU]_{2'OmeR}$-5'
GM-113-6-D'	3'-$[CAGAA]_{2'OmeR}[ACCCCATGAT]_D[ATTCA]_{2'OmeR}$-5'
GM-Control	3'-$[AUCGA]_{2'OmeR}[TACCGTATAA]_D[UAACG]_{2'OmeR}$-5'
**Northern blot**	
Dual DIG-labelled DNA probes	/5Dig/ACCCACGCAGACATATGT/3Dig/
**Methylation primers**	
Mature $tRNA^{Leu}$(TAA) HSA/MMU FW	CCGAGTGGTTAAGGCGTTG
Mature $tRNA^{Leu}$(TAA) HSA/MMU R_D	TGGTACCAGGAGTGG
Mature $tRNA^{Leu}$(TAA) HSA/MMU R_U	GGTACCAGGAGTGGG
pre-$tRNA^{Leu}$(TAA) HSA FW	AAACAAGGTTCAACGTCTGCA
pre-$tRNA^{Leu}$(TAA) HSA R_D	ACCTAAAGCTACCAGGAGTGG
pre-$tRNA^{Leu}$(TAA) HSA R_U	CGAACCCACGCGGACATATG
**qPCR primers**	
Mature $tRNA^{Leu}$(TAA) FW	CCGAGTGGTTAAGGCGTTG
Mature $tRNA^{Leu}$(TAA) RV	CAGGAGTGGGGTTCGAAC
**Taqman Custom designed**	
tRF^{47-64} MMU	UGGACAUAUGUCUGCGUGGGU
tRF^{47-64} HSA	UGGACAUAUGUCCGCGUGGGU
**Housekeeping genes**	
U6– MMU/HSA – FW	AGAAGATTAGCATGGCCCCT
U6– MMU/HSA – RV	ATTTGCGTGTCATCCTTGCG
RPLP0 HSA FW	TCCTCGTGGAAGTGACATCG
RPLP0 HSA RV	TGTCTGCTCCCACAATGAAAC
RPLP0 MMU FW	GTGATGCCCAGGGAAGACAG

RPLP0 MMU RV	TCTGCTCCCACAATGAAGCA

SnoRNAs

SNORD113-6 – FW	TGGACCAGTGATGAATATCATG
SNORD113-6 – RV	TGGACCTCAGAGTTGCAGATG
AF357425 – FW	AGGAGCATGGGGTTTCTGAC
AF357425 – RV	TTTCATAAGGGTTTAATCACTGTCC

Angiogenin

HSA FW	CTGGGCGTTTTGTTGTTGGTC
HSA RV	GGTTTGGCATCATAGTGCTGG
MMU FW	CCAGGCCCGTTGTTCTTGAT
MMU RV	GCAAACCATTCTCACAGGCAATA

COL1A1

HSA FW	TGTGATGGGATTCCCTGGACCTAAAG
HSA RV	TGAGCTCCAGCCTCTCCATCTTTG

ACTA2

HSA FW	AGATCCTGACTGAGCGTGGCTATTC
HSA RV	CTCTTCTCAAGGGAGGATGAGGATGC

Table S2. Expression of tRNA fragments in reads per million (RPM). Small RNA sequencing was performed on primary murine fibroblasts with AF357425 overexpression (High) and knockdown (Low). Fold change is calculated by dividing High by Low RPM. Read counts are quantified in the single assignment approach where reads that map to multiple loci are assigned to the locus that has the highest expression.

antiCodon	High RPM	Low RPM	FoldChange
AlaAGC	342	317	1,08
AlaCGC	169	198	0,85
AlaTGC	334	387	0,86
ArgACG	686	338	2,03
ArgCCG	304	188	1,61
ArgCCT	1000	637	1,57
ArgTCG	786	376	2,09
ArgTCT	235	227	1,04
AsnGTT	446	101	4,40
AspGTC	1109	628	1,77
CysGCA	159	106	1,50
GlnCTG	144	150	0,96
GlnTTG	85	79	1,07
GluCTC	1153	735	1,57
GluTTC	640	489	1,31
GlyACC	22	13	1,67
GlyCCC	167	179	0,94
GlyGCC	817	806	1,01
GlyTCC	322	445	0,72
HisATG	2	2	1,09
HisGTG	213	160	1,33
IleAAT	957	811	1,18
IleGAT	11	7	1,66
IleTAT	20	16	1,20
LeuAAG	256	382	0,67
LeuCAA	255	178	1,43
LeuCAG	272	221	1,23
LeuTAA	238	121	1,97
LeuTAG	157	217	0,72
LysCTT	1758	1357	1,30
LysTTT	1241	1156	1,07
MetCAT	984	437	2,25
PheGAA	250	99	2,53
ProAGG	853	347	2,46

ProCGG	726	355	2,04
ProTGG	2659	1319	2,02
SeC(e)TCA	16	7	2,19
SeCTCA	1	1	0,67
SerAGA	470	406	1,16
SerCGA	77	44	1,75
SerGCT	139	74	1,88
SerGGA	25	8	2,95
SerTGA	161	144	1,12
SupTTA	3	1	2,42
ThrAGT	203	192	1,06
ThrCGT	148	87	1,69
ThrTGT	280	188	1,49
TrpCCA	333	245	1,36
TyrGTA	1610	736	2,19
ValAAC	233	178	1,30
ValCAC	328	261	1,26
ValTAC	88	85	1,04

Table S3. Read counts small RNA sequencing. Small RNA sequencing was performed on PMFs with overexpression (High) or inhibition (Low) of AF357425.

	**High AF357425**	**Low AF357425**
miRbase (sense)	946057,48	951864,44
tRNA	24527,6	16588,28
snoRNA	3167,57	3262,12
Cdna (sense)	2620,37	2459,04
rRNA	2138,41	2084,58
mRNA (antisense)	911,45	845,88
snRNA	96,46	99,15
piRNA	51,18	33,09

Table S4. Absolute Ct values of AF357425 and U6. Small RNA sequencing was performed on primary murine fibroblasts with AF357425 overexpression (3GA-AF25) and knockdown (GM-AF25).

**Sample**	**Ct AF357425**	**Ct U6**
3GA-AF25	21,58	20,65667
GM-AF25	23,71	20,39667

Supplemental Figures

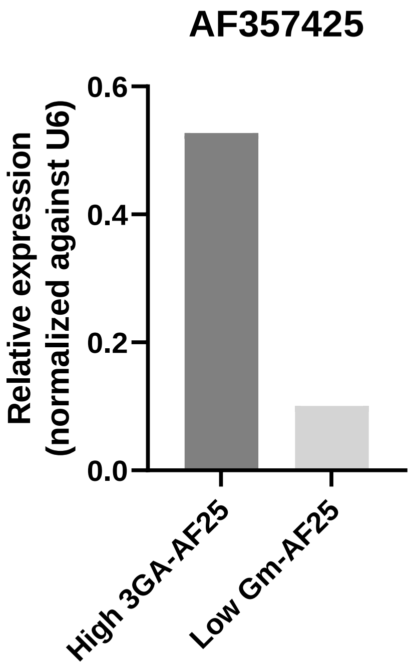

Figure S1. Relative AF357425 expression in small RNA-seq samples. Primary murine fibroblasts were transfected with 3rd Generation Antisense (High 3GA-AF25) or Gapmers (Low Gm-AF25) against AF357425 for 24 hours. Expression levels are normalized to U6.

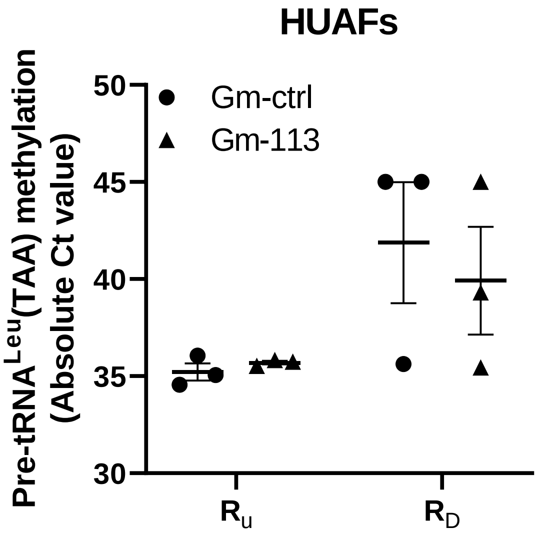

Figure S2. Precursor (pre-)tRNA^{Leu}(TAA) methylation in human arterial fibroblasts (HUAFs). For detection of 2'O-methyated nucleotides, Reversed Transcription at Low dNTP concentration followed by Quantitative PCR (RTL-Q) was performed. Reversed primers were designed upstream (R_U) and downstream (R_D) of the predicted 2'O-methylation (2'Ome) site. RT was performed at low dNTP concentrations. When a 2'Ome site is present, the extension of the R_D primer pauses at this site when low dNTP concentrations are used, whereas the R_U primer does not. Results are shown in absolute Ct values. Ct values above the detection threshold are shown as 45 Ct. N is represented by the individual dots.

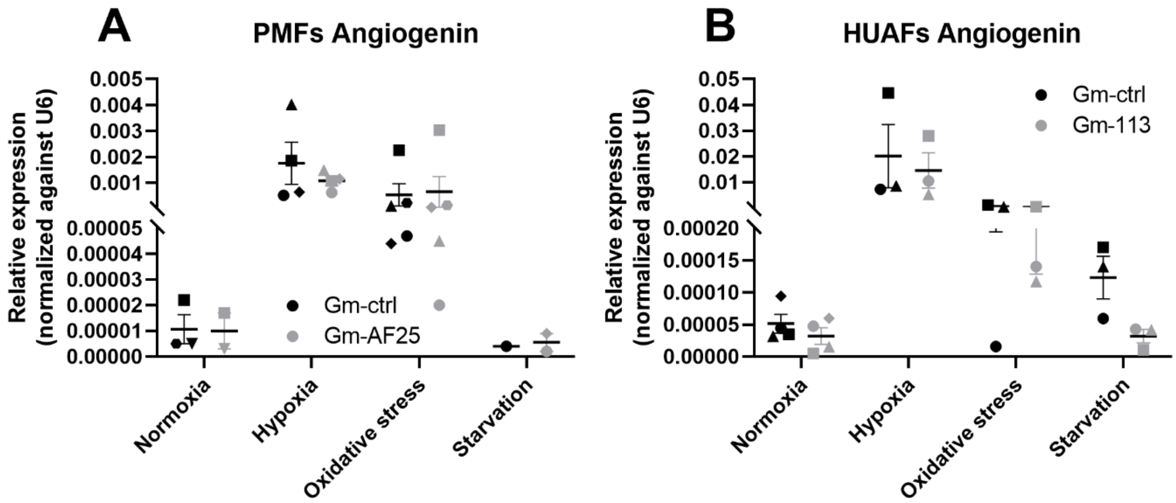

Figure S3. Angiogenin expression in primary cells during cellular stress. (A) Primary murine fibroblasts (PMFs) and (B) human umbilical arterial fibroblasts (HUAFs) were transfected with either Gm-ctrl or Gm-AF25/113 and cultured in normoxic (control), hypoxic, oxidative stress or starvation conditions for 24h. Relative expression levels are normalized to U6. N is represented by the individual dots.

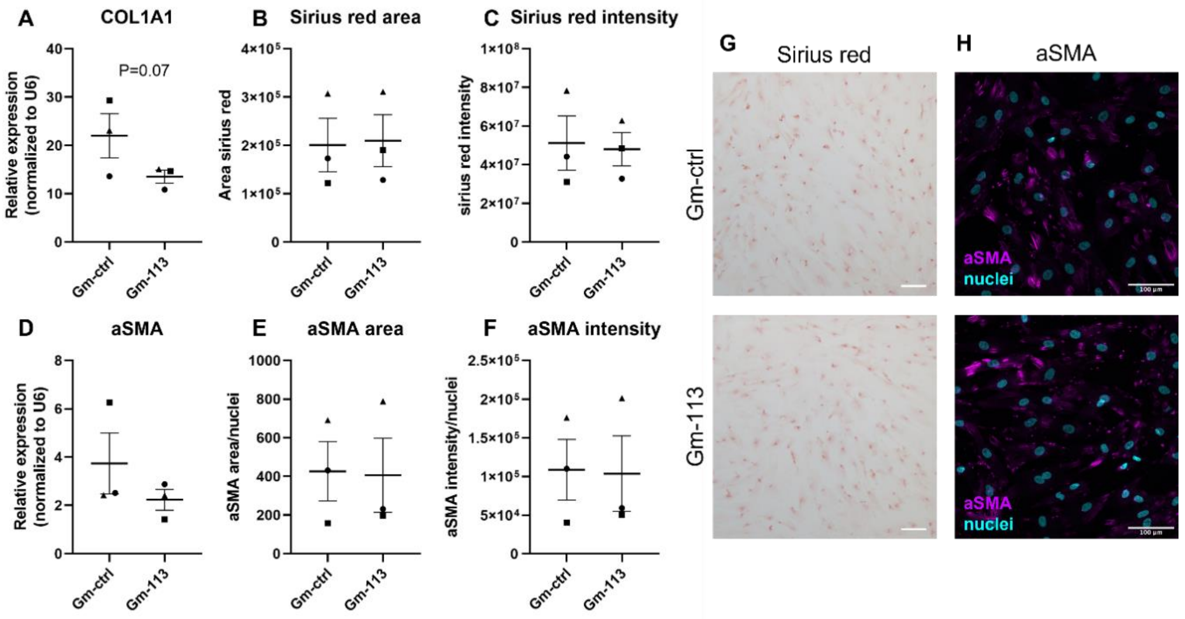

Figure S4. Collagen and alpha-smooth muscle actin (aSMA) expression in human umbilical arterial fibroblasts (HUAFs). (A) Relative expression collagen type 1 alpha 1 chain, (B) area and (C) intensity of Sirius red staining, (D) relative expression of aSMA, (E) area and (F) intensity of aSMA, representative images of (G) Sirius red and (H) aSMA staining of Gm-ctrl and Gm-113 treated HUAFs. HUAFs were treated with Gm-ctrl or Gm-113-6 for 24h. (A, D) Expression levels were normalized to U6. (B,C,D-H) The area was divided by the total amount of nuclei. The integrated density, which is the sum of values of the pixels, was calculated and divided by the total amount of nuclei (intensity). N is represented by the individual dots.

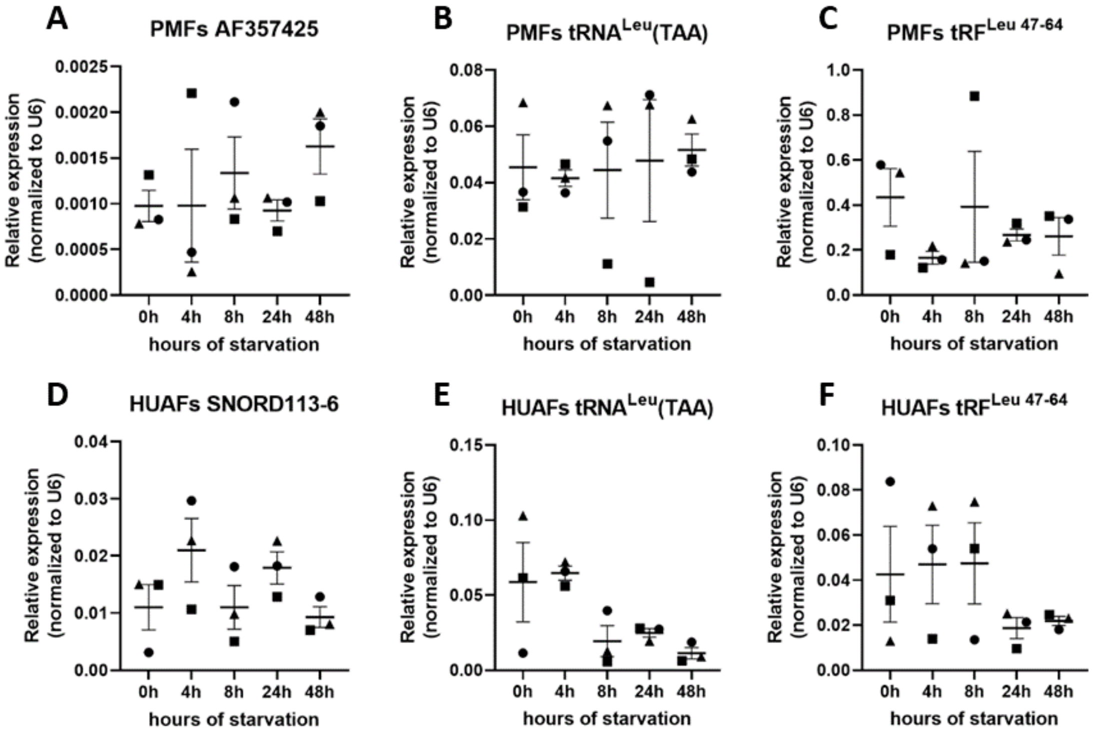

Figure S5. Primary murine fibroblasts (PMFs) and human umbilical arterial fibroblasts (HUAFs) were cultured under different times of serum starvation. Relative expression of (A) AF35742525, (B) tRNA Leucine anti-codon TAA (tRNA^{Leu}(TAA)) and tRNA fragment tRF$^{Leu\ 47\text{-}64}$ in PMFs. Relative expression of (D) SNORD113-6, (E) tRNA^{Leu}(TAA) and (F) tRF$^{Leu\ 47\text{-}64}$ in HUAFs. N is represented by the individual dots.

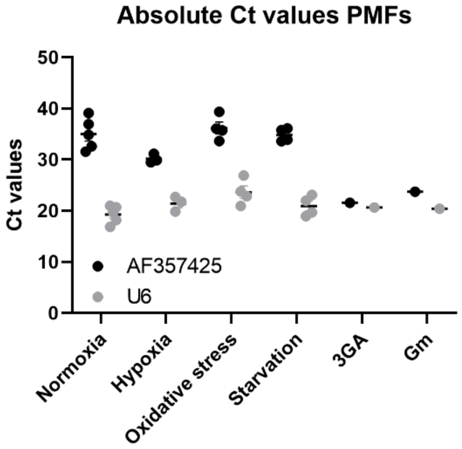

Figure S6. Absolute Ct values of AF357425 and U6 expression in primary murine fibroblasts (PMFs) cultured under different stress conditions or transfected with 3rd Generation Antisense (3GA) or GM for small RNA sequencing. PMFs were cultured under normoxia, hypoxia, oxidative stress or starvation for 24h. Transfection for small RNA sequencing was performed with either 3GA (200 nM) or GM (500 nM) for 24h. N is represented by the individual dots.

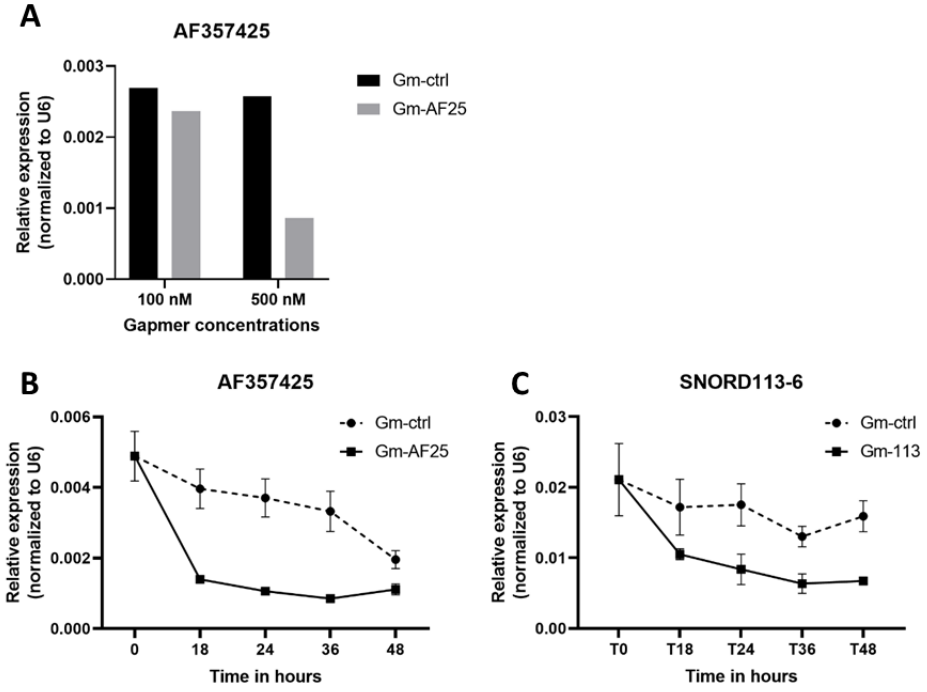

Figure S7. Optimization of GM transfection. Two concentrations of GM and different times of transfection were tested for optimal inhibition. (A) Primary murine fibroblasts (PMFs) were transfected with a concentration of 100 or 500 nM GM against AF357425 (GM-AF25) or a control (GM-ctrl) for

24h. Different times of transfection against (B) AF357425 in PMFs and (C) SNORD113-6 in HUAFs were tested (N=3). (B,C) Relative expression levels are normalized to U6.

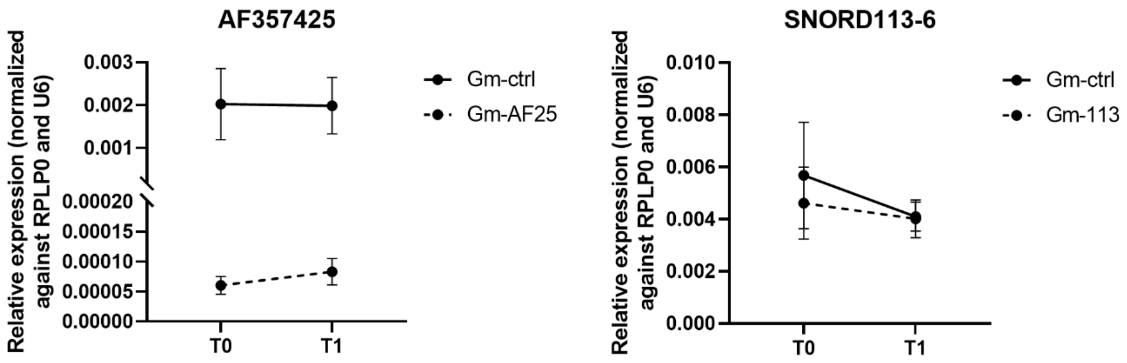

Figure S8. Relative AF357425 and SNORD113-6 expression in PMFs and HUAFs, respectively, before and after 1h Actinomycin D treatment. Cells were first transfected with GM against AF357425 (Gm-AF25) or SNORD113-6 (Gm-113), or a control (Gm-ctrl), and then treated with Actinomycin D. Expression levels are normalized to RPLP0 and U6.
