# Supplementary material for: C/D box snoRNA SNORD113-6 guides 2′-O-methylation and protects against site-specific fragmentation of tRNALeu(TAA) in vascular remodeling
Source: Mol Ther Nucleic Acids. 2022 Sep 17;30:162–72. doi: 10.1016/j.omtn.2022.09.011 (PMC9547152; doi:10.1016/j.omtn.2022.09.011)
Supplement: Document S2. Article plus supplemental information [file mmc2.pdf]

Molecular Therapy
Nucleic Acids
Original Article

C/D box snoRNA SNORD113-6 guides 2′-O-methylation and protects against site-specific fragmentation of tRNA^{Leu}(TAA) in vascular remodeling

Eva van Ingen,[1,2] Pleun A.M. Engbers,[1,2] Tamar Woudenberg,[1,2] M. Leontien van der Bent,[1,2] Hailiang Mei,[3] Johann Wojta,[4,6] Paul H.A. Quax,[1,2] and A. Yaël Nossent[1,2,4,5]

[1]Department of Surgery, Leiden University Medical Center, PO Box 9600, 2300 Leiden, the Netherlands; [2]Einthoven Laboratory for Experimental Vascular Medicine, Leiden University Medical Center, Leiden, the Netherlands; [3]Department of Biomedical Data Sciences, Leiden University Medical Center, Leiden, the Netherlands; [4]Department of Internal Medicine II, Medical University of Vienna, Vienna, Austria; [5]Department of Laboratory Medicine, Medical University of Vienna, Vienna, Austria; [6]Ludwig Boltzmann Institute for Cardiovascular Research, Vienna, Austria

C/D box small nucleolar RNAs (snoRNAs) of the DLK1-DIO3 locus are associated with vascular remodeling and cardiovascular disease. None of these snoRNAs has any known targets yet except for one, AF357425/SNORD113-6. We previously showed that this snoRNA targets mRNAs of the integrin signaling pathway and affects arterial fibroblast function. Here, we aimed to identify whether AF357425/SNORD113-6 can also target small RNAs. We overexpressed or inhibited AF357425 in murine fibroblasts and performed small RNA sequencing. Expression of transfer (t)RNA fragments (tRFs) was predominantly regulated. Compared with overexpression, AF357425 knockdown led to an overall decrease in tRFs but with an enrichment in smaller tRFs (<30 nucleotides). We focused on tRNA leucine anti-codon TAA (tRNA^{Leu}(TAA)), which has a conserved predicted binding site for AF357425/SNORD113-6. Adjacent to this site, the tRNA is cleaved to form tRF$^{Leu\ 47–64}$ in both primary murine and human fibroblasts and in intact human arteries. We show that AF357425/SNORD113-6 methylates tRNA^{Leu}(TAA) and thereby prevents the formation of tRF$^{Leu\ 47–64}$. Exposing fibroblasts to oxidative or hypoxic stress increased AF357425/SNORD113-6 and tRNA^{Leu}(TAA) expression, but AF357425/SNORD113-6 knockdown did not increase tRF$^{Leu\ 47–64}$ formation under stress even further. Thus, independent of cellular stress, AF357425/SNORD113-6 protects against site-specific fragmentation of tRNA^{Leu}(TAA) via 2′O-ribose-methylation.

INTRODUCTION

Vascular remodeling is the collective name for both adaptive and maladaptive changes to the vessel wall. This includes processes like angiogenesis and arteriogenesis on the one hand and atherosclerosis and aneurysm formation on the other.[1] Vascular remodeling is the predominant underlying cause of most cardiovascular disease. All layers of the arterial wall, i.e., the tunica intima made up of endothelial cells, the tunica media made up of smooth muscle cells, and the tunica adventitia made up of predominantly fibroblasts, play their own role in vascular remodeling, but the role of the adventitial fibroblasts is often underestimated.[2] Although cardiovascular disease has a complex pathology, ischemia plays an intricate part in both the development and manifestation of cardiovascular disease. Ischemia induces several forms of cellular stress, including nutrient deprivation and hypoxia, that have all been linked to increases in posttranscriptional modifications of RNA.[3–7]

Small nucleolar RNAs (snoRNAs) are a type of small noncoding RNA that mediate RNA modifications at the post-transcriptional level. There are two types of snoRNAs, C/D box and H/ACA box snoRNAs, named after their conserved sequence motifs. C/D box snoRNAs guide 2′-O-ribose methylation (2′Ome) of their target RNAs. The DLK1-DIO3 locus on the long arm of human chromosome 14 encodes a cluster of 41 C/D box snoRNAs (14q32; 12F1 in mice). We have demonstrated that this cluster of 14q32 C/D box snoRNAs is strongly associated with vascular remodeling and human cardiovascular disease.[8–10] The association with cardiovascular disease is both independent of and stronger than the 14q32 long noncoding RNAs (lncRNAs) and the cluster of 14q32 microRNAs that lie adjacent to the snoRNA genes.[8] Furthermore, plasma levels of 14q32 snoRNAs were associated with disease outcome in patients with peripheral arterial disease (PAD).[9,10] However, all 14q32 C/D box snoRNAs, except for one, are orphan snoRNAs, meaning that they have no known RNA targets.

We recently demonstrated that one of the most abundantly expressed snoRNAs of the 14q32 cluster, human SNORD113-6, and its murine equivalent, AF357425, target mRNAs of the integrin signaling pathway, influencing both pre-mRNA processing and 2′Ome. The D’ antisense box of AF357425/SNORD113-6 is fully conserved

Received 23 December 2021; accepted 15 September 2022;
https://doi.org/10.1016/j.omtn.2022.09.011.

Correspondence: A. Yaël Nossent, PhD, Department of Surgery, Leiden University Medical Center, PO Box 9600, 2300 Leiden, the Netherlands.
E-mail: a.y.nossent@lumc.nl

This is an open access article under the CC BY license (http://creativecommons.org/licenses/by/4.0/).

between humans and mice. Fibroblast integrin signaling is important for cell-cell and cell-matrix interactions and acts in various forms of cardiovascular remodeling that can lead to cardiovascular disease.[11] Indeed, knockdown of SNORD113-6 altered human arterial fibroblast function.[12]

C/D box snoRNAs associate with four conserved ribonucleoproteins NHP2L1, NOP56, NOP58, and Fibrillarin (FBL). FBL is the methyltransferase that catalyzes 2′Ome. C/D box snoRNAs have two antisense boxes, located directly upstream of the D and D' boxes, which are not covered by ribonucleoproteins and are thus free to interact with target RNA sequences. C/D box snoRNAs hybridize to their target RNAs via Watson-Crick base-pairing. Once bound to the target RNA, the 5th nucleotide upstream of the D or D' box is positioned for 2′Ome.[13–15] Many expressed C/D box snoRNAs, however, lack antisense elements of known rRNA 2′Ome sites and are considered orphan snoRNAs.[16] Likely, these orphan C/D box snoRNAs target other types of RNA molecules than rRNA.[17–19]

Besides rRNAs, transfer (t)RNAs are the most heavily modified cellular RNAs.[20] Their canonical function lies in protein translation, where they deliver amino acids to the translating peptide chain. However, recent reports show that tRNAs can be processed into tRNA-derived fragments (tRFs), which can perform other, noncanonical, functions. tRFs can derive from different regions of their parental tRNA, located anywhere from the 5′ to 3′ end, and have variable sizes up to ∼50 nucleotides.[21,22] Fragmentation of tRNAs can be induced under cellular stress, such as oxidative stress and hypoxia, which are important triggers of vascular remodeling processes.[23–25] Among others, angiogenin (ANG) is a tRNA-processing endonuclease that is activated during cellular stress.[23]

Recent findings show that RNA modifications guided by snoRNAs can prevent tRNA cleavage and thereby regulate tRF formation.[21,26–29] For instance, SNORD97 induces 2′Ome on the wobble cytidine C34 of tRNA^{Met}(CAT), which protects against ANG-induced cleavage.[26] Whether AF357425/SNORD113-6 also guides 2′Ome on small RNA molecules like tRNAs, however, is still unknown.

Here, we aimed to determine whether AF357425/SNORD113-6 can target small RNAs in the vasculature, using primary murine fibroblasts (PMFs) and primary human umbilical arterial fibroblasts (HUAFs). Fibroblasts were chosen for their relevance in vascular remodeling[2] and because the 14q32 snoRNAs are expressed most abundantly in fibroblasts.[16] We used both murine and human fibroblasts to be able to look only at putative target RNAs that were conserved between the two species. We confirmed our key findings using an *ex vivo* model for ischemia in intact human arteries.[30] We performed small RNA sequencing (sRNA-seq) on primary PMFs in which we either inhibited or overexpressed AF357425. We found that tRFs were the predominant group of small RNAs that changed in expression. Knockdown of AF357425 resulted in an apparent reduction of total tRFs but an enrichment of smaller sized tRFs (18–30 nucleotides). We focused on one of these tRNAs, tRNA leucine anti-codon TAA (tRNA^{Leu}(TAA)), which has a predicted binding site for AF357425 in mice and SNORD113-6 in human. sRNA-seq data showed that its dominant tRF, tRF$^{Leu\ 47–64}$, is formed just upstream of this site. Formation of tRF$^{Leu\ 47-–64}$ was conserved in both PMFs and HUAFs and was investigated under oxidative, hypoxic, and starvation stress. We show that AF357425/SNORD113-6 indeed methylates this tRNA and protects against site-specific tRNA^{Leu}(TAA) fragmentation.

RESULTS

sRNA-seq in PMFs

In order to identify small RNA targets of AF357425/SNORD113-6, we performed sRNA-seq on PMFs in which we either inhibited or overexpressed AF357425. With this strategy, we aimed to obtain the largest possible difference in small RNA target expression. Gapmers were used to inhibit AF357425 expression (GM-AF25) and 3rd generation antisense oligonucleotides (3GAs) to overexpress AF357425 (3GA-AF25). We showed previously that 3GAs directed against the 3′ end of AF357425 (3GA-AF25) induced snoRNA overexpression, likely through protection from degradation by endonucleases.[8] Expression of AF357425 was increased (>5-fold) in PMFs treated with 3GA-AF compared with GM-AF25 (Figure S1). By far, most reads from the sRNA-seq in both samples were mapped to microRNAs. Read counts mapped to microRNAs, as well as those that mapped to the much lower expressed snoRNA and rRNA genes, were similar between AF25-high and AF25-low cells. However, reads that mapped to tRNA genes, which are all tRFs of <45 nucleotides in length, appeared to be reduced in number in AF25-low cells (Figure 1A; Tables S2 and S3). Where the longer tRFs (30–45 nucleotides) appeared enriched in AF25-high cells, smaller-sized tRFs (18–29 nucleotides) were enriched in AF25-low cells, particularly the 18-nucleotide tRFs (Figure 1B). These data indicate that AF357425 may protect against tRNA fragmentation.

Fragmentation of tRNA^{Leu}(TAA)

In order to investigate the mechanisms through which AF357425/SNORD113-6 may influence tRNA fragmentation, we focused on a single tRNA. Among others, tRNA^{Leu}(TAA) had a predicted D' box antisense sequence for AF357425. This site was conserved in human tRNA^{Leu}(TAA) for the D' box antisense sequence of SNORD113-6. Except for the outer 2 nucleotides, a perfect reverse complementary sequence for the middle 7 nucleotides is present in both mouse and human tRNA^{Leu}(TAA) (AACCCCA; Figure 2B). A tRF cleaved just upstream of this predicted 2′Ome site (18–20 nucleotides; Figure 2A), tRF$^{Leu\ 47–64}$, was abundantly expressed in both AF25-high and AF25-low cells (Figure 2B). However, the total tRFs generated from tRNA^{Leu}(TAA) were decreased in AF25-low cells. In contrast, tRF$^{Leu\ 47–64}$ was more abundant relative to the total tRFs in AF25-low (50%) compared with AF25-high cells (35%; Figures 2B and 2C). We confirmed expression of tRF$^{Leu\ 47–64}$ by northern blot in both PMFs and in HUAFs. Expression of tRF$^{Leu\ 47–64}$ appeared enhanced under oxidative stress (Figures 2D and 2E).

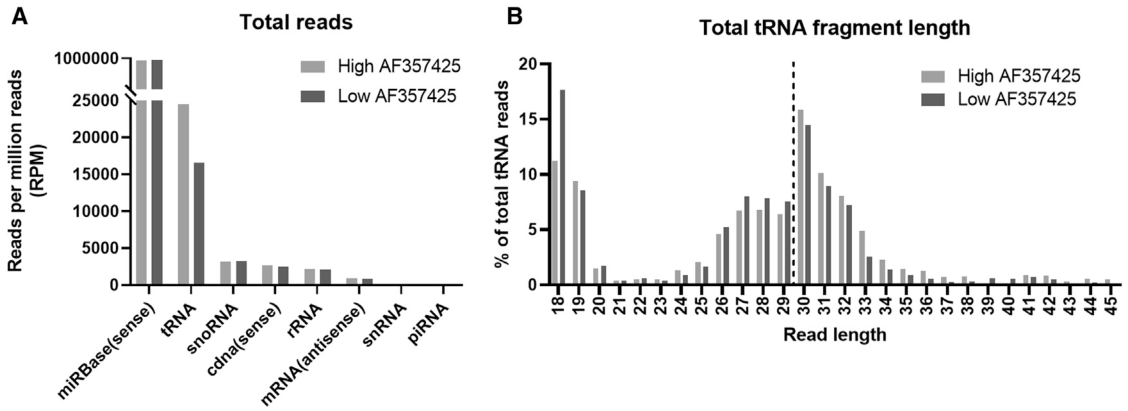

Figure 1. sRNA-seq

(A) Total reads mapped to small RNAs, normalized to reads per million (RPM) and (B) total tRNA fragment length (in nucleotides), shown as a percentage (%) of total tRNA reads in high AF357425 (overexpressed) and low AF357425 (inhibited) primary murine fibroblasts. N.B., Piwi-interacting RNAs (piRNAs) and small nuclear RNAs (snRNAs) were detected, but the number of reads was too low to be visible in the graph at this y-axis scale.

Validation of tRNA^{Leu}(TAA) 2′Ome

Next, we performed reverse transcription at low dNTP concentration followed by quantitative PCR (RTL-Q) to calculate the estimated methylated fraction (EMF), using site-specific primers for detection of 2′Ome. We confirmed 2′Ome of the mature full-length tRNA^{Leu}(-TAA), located on the 5th nucleotide upstream of the D' antisense box, in both PMFs and HUAFs. Inhibition of AF357425/SNORD113-6 partly reduced 2′Ome at this site compared with a gapmer control (GM-ctrl; p for trend = 0.0725 in PMFs and 0.0968 in HUAFs; Figure 3). 2′Ome also appeared present on the precursor-(pre)tRNA^{Leu}(-TAA) in HUAFs, but we could not confirm snoRNA-induced regulation of 2′Ome in the pre-tRNA^{Leu}(TAA) due to high Ct values above the detection threshold (>45 Ct; Figure S2). The validation of 2′Ome in both mouse and human cells suggests that both are evolutionarily conserved features of tRNA^{Leu}(TAA).

Fragmentation of tRNA^{Leu}(TAA) under cellular stress

As fragmentation of tRNAs can be induced during cellular stress,[23–25] we cultured PMFs and HUAFs under different cellular stress conditions and measured expression levels of AF357425/SNORD113-6, mature tRNA^{Leu}(TAA), and tRF$^{Leu\ 47-64}$ by qPCR. Endogenous expression of AF357425/SNORD113-6 and mature tRNA^{Leu}(TAA) showed similar expression patterns in PMFs and HUAFs, with increased expression under both hypoxia and oxidative stress, compared with the normal culture condition control. Serum starvation, on the other hand, did not induce changes in either AF357425/SNORD113-6 or tRNA^{Leu}(TAA) expression compared with normal culture conditions. Expression of tRF$^{Leu\ 47-64}$ was only increased significantly under hypoxia in both PMFs and HUAFs but appeared slightly elevated under oxidative stress as well (Figure 4).

Subsequently, PMFs and HUAFs were transfected with either GM-AF25/113 or a GM-ctrl and were cultured under the different cell stress conditions. The absolute Ct value of mature tRNA^{Leu}(TAA) was divided by the Ct value of tRF$^{Leu\ 47-64}$ in order to quantify the expression of tRF$^{Leu\ 47-64}$ relative to mature tRNA^{Leu}(TAA), which it was generated from. The ratio was increased under AF357425/SNORD113-6 inhibition under control conditions in both PMFs and HUAFs (Figure 5). In PMFs, the ratio was also increased under hypoxia and showed a trend toward an increased ratio under serum starvation (Figure 5A). This increased ratio demonstrates that more tRF$^{Leu\ 47-64}$ is formed relative to its mature tRNA, when AF357425/SNORD113-6 is inhibited. When we quantified expression of ANG, we did not observe a difference between GM-AF25/113 and GM-ctrl (Figure S3). However, ANG did increase under cellular stress, similar to the snoRNA, both in PMFs and HUAFs. We can neither confirm nor exclude that ANG is responsible for cleavage of tRNA-^{Leu}(TAA), but we can conclude that ANG is not influenced by the snoRNA directly and that changes in fragmentation are likely caused by changes in snoRNA-guided 2′Ome of the tRNA.

snoRNA, tRNA, and tRF expression in human arteries

In many vascular pathologies, adventitial fibroblasts are activated and become myofibroblasts.[2] We therefore examined whether inhibition of SNORD113-6 affects this phenotype transition (Figure S4). There was a trend toward reduced collagen type 1 alpha 1 chain (COL1A1) expression in GM-113-treated fibroblasts (p = 0.07); however, protein expression was affected by snoRNA inhibition. Although α-smooth muscle actin (αSMA) was expressed, as is common for fibroblasts in culture, we did not observe differences between GM-113- and GM-ctrl-treated cells.

To examine whether tRNA^{Leu}(TAA) fragmentation increases under stress conditions in human vascular tissues, we used internal mammary arteries (IMAs), which were harvested during elective coronary bypass surgery on patients with coronary artery disease. After culturing the vessels *ex vivo* under control or ischemic (= hypoxia + starvation) conditions for 24 h, expressions of SNORD113-6, tRNA^{Leu}(TAA), and tRF$^{Leu\ 47-64}$ were measured. Consistent with our *in vitro* models,

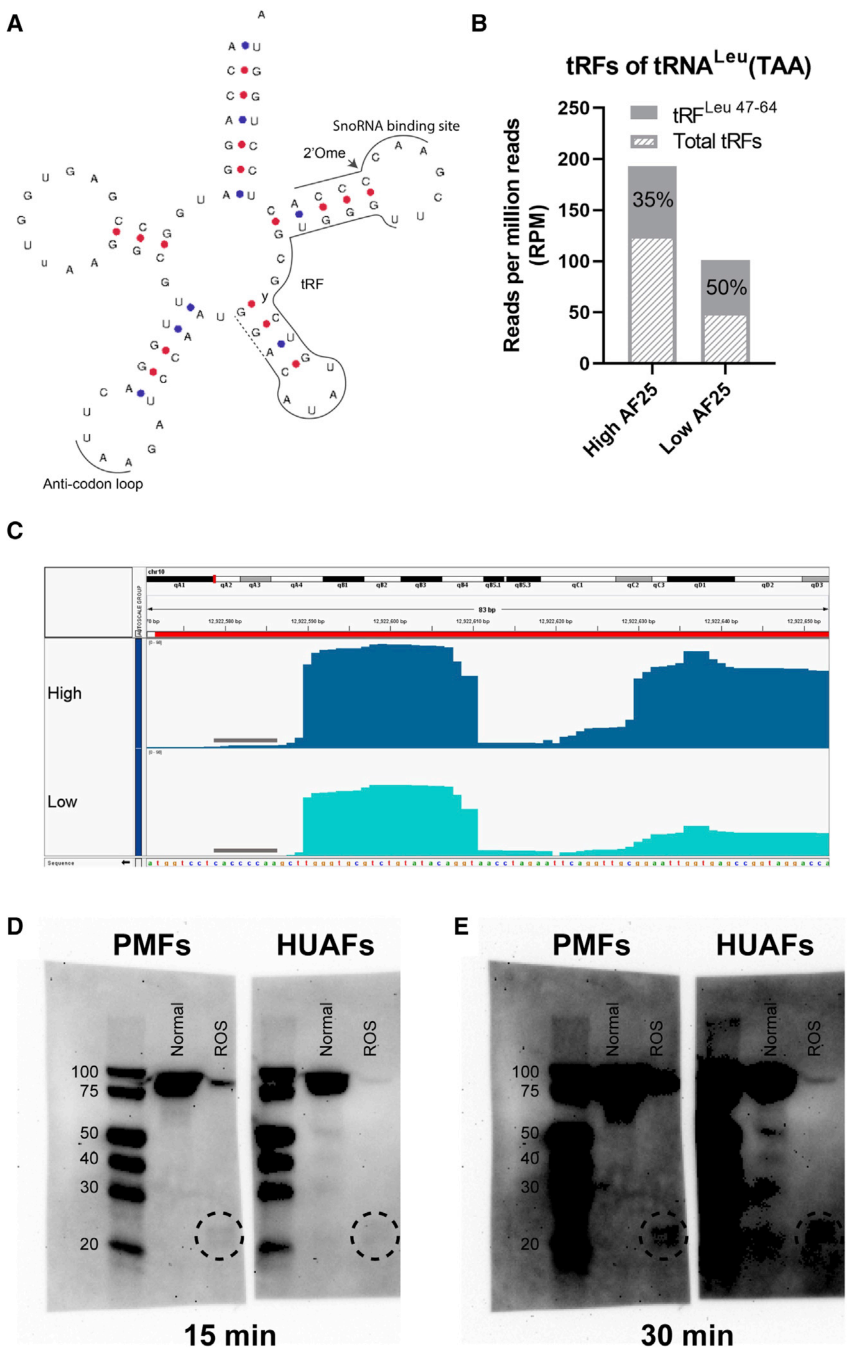

Figure 2. Fragmentation of tRNA^{Leu}(TAA)

(A) Schematic overview of the mature tRNA^{Leu}(TAA) sequence in mouse and human. The AF357425/SNORD113-6 predicted binding site (snoRNA binding site), tRNA fragment generated upstream of the binding site, tRF$^{Leu\ 47–64}$, and the anti-codon loop are indicated with a line. The dashed line indicates length extension of tRF$^{Leu\ 47–64}$, representing 3 different sizes of generated tRFs. Arrow points at the predicted 2′O-methylation (2′Ome) site. γ represents U in mice and C in human. Figure adapted from GtRNAdb.[31] (B) The fragment of interest, tRF$^{Leu\ 47–64}$, shown as a percentage of total tRFs generated from tRNA^{Leu}(TAA). Data are normalized to RPM. (C) Coverage plot of tRNA^{Leu}(TAA) in primary murine fibroblasts with either high or low AF357425 expression. The predicted AF357425 binding site is indicated with a line. Sequence is shown from 3′ to 5′. (D and E) Northern blots exposed for (D) 15 and (E) 30 min using the ChemiDoc-IT imaging system. Primary murine fibroblasts (PMFs) and human umbilical arterial fibroblasts (HUAFs) were cultured in normoxic and reactive oxidative stress (ROS) conditions. A digoxigenin labeled probe was used to visualize tRFs. A circle indicates tRF$^{Leu\ 47–64}$.

expression of SNORD113-6 was significantly upregulated ($p = 0.0432$) under ischemic conditions (Figure 6A). tRNA^{Leu}(TAA) also appeared somewhat upregulated, but not significantly (Figure 6B). tRF$^{Leu\ 47–64}$ expression, however, was not altered (Figure 6C). When we calculated the Ct tRNA^{Leu}(TAA)/tRF$^{Leu\ 47–64}$, there was a trend toward a decrease in the ratio, indicating that less tRNA^{Leu}(TAA) was cleaved into tRF$^{Leu\ 47–64}$ (Figure 6D).

Effects of 2′Ome on tRNA stability

Ribonucleotide modifications in the structural core of the tRNA may stabilize the tRNA and reduce tRNA degradation rates.[32] After transfection with either GM-AF25/113 or GM-ctrl, cells were treated with a high concentration of actinomycin D (5 μg/μL) for 1 h to inhibit novel tRNA transcription. In both PMFs and HUAFs, mature tRNA^{Leu}(TAA) was rapidly degraded, but no differences were observed between GM-AF25/113 and GM-ctrl (Figures 7A and 7C). In PMFs, the relative expression of mature tRNA^{Leu}(TAA) was lower to begin with in AF25-low cells and remained lower after 1 h, compared with GM-ctrl (Figure 7A). To rule out differences in degradation rates of housekeeping genes used (RPLP0 and U6) between the two groups, we also normalized the expression levels at 1 to 0 h (T0; Figures 7B and 7D). Indeed, no differences in degradation rates between GM-AF25/113 or GM-ctrl were observed, indicating that a reduction of this single 2′Ome modification did not affect overall mature tRNA^{Leu}(TAA) stability but only the site-specific fragmentation.

DISCUSSION

Here, we aimed to further elucidate the role of the vasoactive 14q32 snoRNA AF357425/SNORD113-6 in vascular remodeling. We investigated whether AF357425/SNORD113-6 could also target small RNAs

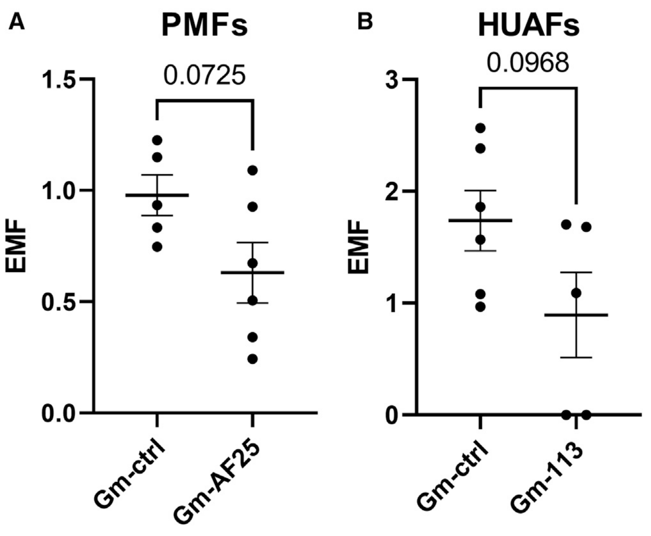

Figure 3. 2′Ome at predicted AF357425/SNORD113-6 binding sites on $tRNA^{Leu}$(TAA)

For detection of 2′O-methyated nucleotides and to calculate the estimated methylated fraction (EMF), reversed transcription at low dNTP concentration followed by quantitative PCR (RTL-Q) was performed. Site-specific reversed primers were used exactly on and 1 nucleotide downstream of the predicted 2′Ome site to accurately determine the exact location of the 2′Ome site. (A) PMFs or (B) HUAFs were transfected with gapmers against AF357425 (GM-AF25)/SNORD113-6 (GM-113) or a gapmer control (GM-ctrl) for 24 h. Data represent a minimum of three independent experiments. n is represented by the individual dots. Data are represented as mean ± SEM. A two-tailed unpaired t test was performed to compare GM-AF25/113 with GM-ctrl.

besides the previously identified integrin signaling mRNA targets.[33] We found that tRNAs are the predominant small RNA target of AF357425 in primary fibroblasts. Inhibition of AF357425/SNORD113-6 led to an overall decrease in tRFs, and, compared with overexpression of the snoRNA, less larger (>30 nucleotides) and more smaller (<30 nucleotides) tRFs were formed. In order to investigate the underlying mechanisms of action, we focused on $tRNA^{Leu}$ (TAA), which has a conserved binding site for the D' box seed sequence of AF357425/SNORD113-6. We showed that $tRNA^{Leu}$(TAA) is a 2′Ome target of AF357425/SNORD113-6 and that snoRNA inhibition led to an apparent reduction of 2′Ome at this site, both in murine and in human primary fibroblasts. Endogenous expression of AF357425/SNORD113-6 and mature $tRNA^{Leu}$(TAA) both increased under hypoxia and oxidative stress. Endogenous $tRF^{Leu\ 47-64}$ expression was also elevated under hypoxia *in vitro*. In intact human arteries however, ischemia-induced upregulation of SNORD113-6 appeared to reduce $tRNA^{Leu}$(TAA) fragmentation into $tRF^{Leu\ 47-64}$. Knockdown of AF357425/SNORD113-6 resulted in an increased ratio of $tRF^{Leu\ 47-64}$ relative to its mature $tRNA^{Leu}$(TAA). 2′Ome by AF357425/SNORD113-6 was not important for the overall stability of the tRNA, and therefore, we conclude that it acts via protecting against site-specific fragmentation of $tRNA^{Leu}$(TAA) into $tRF^{Leu\ 47-64}$.

Regarding the biological role of $tRF^{Leu\ 47-64}$ in vascular remodeling and cardiovascular disease, much remains unclear. Just like we show here, several other recent studies have shown that fragmentation of tRNAs increases under cellular stress.[23–25,34] Similar cellular stress conditions also trigger vascular remodeling. This implicates that the formation of tRFs play a regulatory role in vascular remodeling. Indeed, oxygen-glucose deprivation, which is an *in vitro* model for ischemic reperfusion injury, induced tRNA cleavage in neuronal cells.[25] Also *in vivo*, in models for acute ischemic stroke and PAD, the formation of tRFs was strongly increased.[24] Similarly, we have previously shown that the 14q32 snoRNAs are regulated under ischemic conditions in patients with PAD and during vascular remodeling.[8] In the current study, we show that expression of AF357425/SNORD113-6 indeed increased during hypoxia and oxidative stress in cells, as well as under ischemia in whole human inner mammary arteries cultured *ex vivo*. In whole artery tissue, ischemia appear to reduce the cleavage of $tRNA^{Leu}$(TAA) into $tRF^{Leu\ 47-64}$ when looking at their Ct ratio. In cells, we did not observe this decrease; however, exposing cells to cellular stress did not result in an additional increase of $tRF^{Leu\ 47-64}$ to mature $tRNA^{Leu}$(TAA) ratio in AF357425/SNORD113-6 knockdown cells, whereas expression of mature $tRNA^{Leu}$(TAA) and ANG both increased under both hypoxia and oxidative stress.

Even though our findings implicate a role for site-specific fragmentation of tRNAs in vascular remodeling, the question remains what the molecular and biological function of the formed tRNA fragment $tRF^{Leu\ 47-64}$ could be. Assumingly, $tRF^{Leu\ 47-64}$ has an important role in vascular biology, as $tRF^{Leu\ 47-64}$ is also generated under physiological conditions and not exclusively during cellular stress. Our group, as well as others, have shown that tRFs have potential as circulating biomarkers in, for example, acute stroke.[35–37] It has also been demonstrated that tRFs can perform all sorts of regulatory functions, including regulation of protein translation, microRNA-like functions by base-pairing with mRNAs, and interaction with RNA-binding proteins.[38,39] Furthermore, tRFs have been shown to be functionally active in modulating cardiac and skeletal muscle function and endothelial function but also in inhibition of angiogenesis.[24,40] Which exact regulatory function(s) $tRF^{Leu\ 47-64}$ may have and how it impacts vascular function, as well as vascular remodeling, remains to be determined.

Regarding the molecular role of snoRNA-induced 2′Ome of $tRNA^{Leu}$(TAA), the same post-transcriptional modifications on tRNAs have been shown to both protect from and promote fragmentation.[27,28] Our data suggest that AF357425/SNORD113-6 2′Ome protects the tRNA from cleavage into small fragments (∼18 nucleotides in length) rather than promoting it. However, we found more tRFs in total, including all tRFs formed of $tRNA^{Leu}$(TAA), and longer fragments (>30 nucleotides) in AF357425-high cells than in AF357425-low cells. Perhaps the presence or absence of modifications attracts different tRNA endonucleases, which produce different tRF species. The role of AF357425/SNORD113-6 2′Ome in cleavage of other tRNAs remains to be determined, but likely, its function is to prevent fragmentation of shorter tRFs (∼18 nucleotides in length).

Besides the stabilizing tertiary structure, little is known about other functions of post-transcriptional modifications in the structural

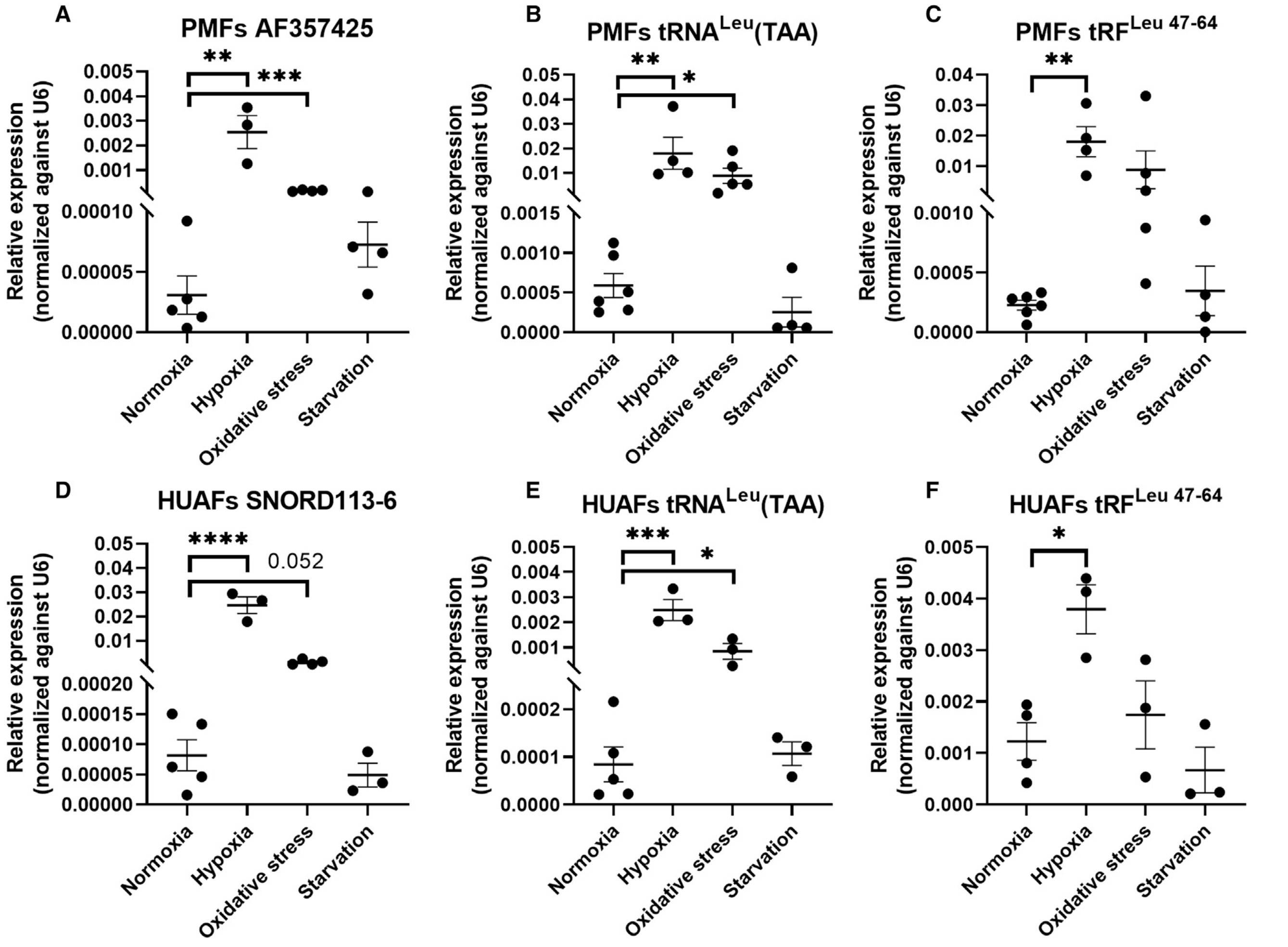

Figure 4. Relative snoRNA, tRNA^{Leu}(TAA), and tRF$^{Leu\ 47–64}$ expression during cellular stress

Relative expression levels in (A–C) PMFs and (D–F) HUAFs cultured in normoxic, hypoxic, oxidative stress, and starvation conditions. Expression levels are normalized to U6. Data represent a minimum of three independent experiments. n is represented by the individual dots. Data are represented as mean ± SEM. A two-tailed unpaired t test was performed to compare treatment with the control. *p < 0.05, **p < 0.01, ***p < 0.005, compared with normoxia.

core of tRNAs.[32] AF357425/SNORD113-6 targets and guides 2′Ome in the T-arm, the structural core, of tRNA^{Leu}(TAA). 2′Ome and the formation of tRF$^{Leu\ 47–64}$ were found in both mice and humans, suggesting that both are evolutionarily conserved features of tRNA^{Leu}(-TAA). Degradation rates of tRNA^{Leu}(TAA) were similar between GM-ctrl and GM-AF25/113 transfected cells, indicating that 2′Ome by AF357425/SNORD113-6 is not important for the overall tRNA-^{Leu}(TAA) stability. Of course, tRNAs are heavily modified, and reduction of a single 2′Ome modification may not have direct consequences for their stability. This could however pose a threat to the reliability of our measurements. Modifications on tRNAs may impede reverse transcription and limit detection of tRFs and tRNAs by qRT-PCR.[41] However, 2′Ome by AF357425/SNORD113-6 is located toward the 3′ end of the tRNA. We designed qPCR primers for mature tRNA^{Leu}(TAA) upstream of that site in order to limit confounding effects by the presence or absence of 2′Ome. Furthermore, expression of tRF$^{Leu\ 47–64}$, which we initially found in the sRNA-seq, was confirmed by northern blot, and our qPCR results showed that tRF$^{Leu\ 47–64}$ and mature tRNA^{Leu}(TAA) were both abundantly expressed. We cannot control for effects of other modifications in our qPCRs; however, if reversed transcription was hampered by other modifications, these were likely similar between GM-AF25/113 and GM-ctrl.

Taken together, we show that AF357425/SNORD113-6 targets predominantly tRNAs, protecting the tRNA from cleavage into small fragments. When zooming in on one specific tRNA, tRNA^{Leu}(TAA), we show that AF357425/SNORD113-6 induces 2′Ome of the mature tRNA, thereby protecting against site-specific tRNA fragmentation. The function of tRF$^{Leu\ 47–64}$ in vascular remodeling and whether this tRF forms a potential future therapeutic target for treatment and/or prevention of cardiovascular disease, however, remain to be elucidated.

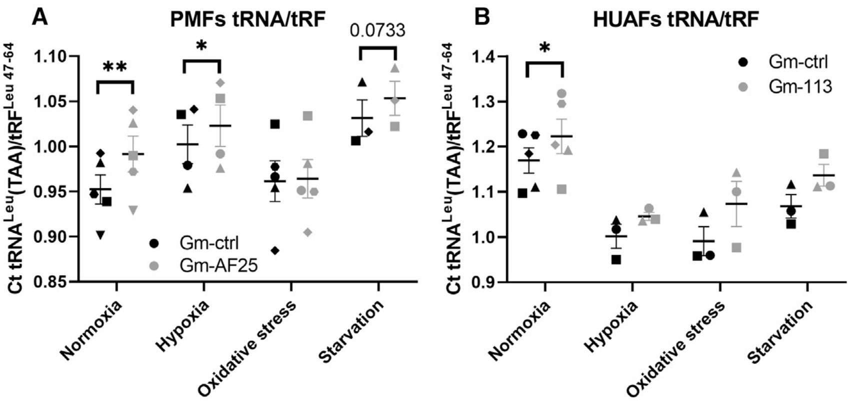

Figure 5. Ratio mature tRNA^{Leu}(TAA)/tRF$^{Leu\ 47–64}$ in primary cells exposed to different cell stress stimuli
(A) PMFs and (B) HUAFs transfected with GM-AF25/113 or GM-ctrl and cultured in normoxic, hypoxic, oxidative stress, or starvation conditions. Expression levels were measured by qPCR. Ratios were calculated by dividing absolute Ct values of the mature tRNA by Ct values of the tRF. Data represent a minimum of three independent experiments. n is represented by the individual dots. Data are represented as mean ± SEM. A two-tailed paired t test was performed to compare single treatment with the control, within each experiment. *$p < 0.05$, **$p < 0.01$, compared with GM-ctrl.

MATERIALS AND METHODS

Cell culture

Cells were cultured in a humidified incubator at 37°C under 5% CO_2. Cells were passaged at 70%–90% confluency and used up to passage 6. DMEM, supplemented with 10% heat-inactivated fetal calf serum (FCSi) and 1% Pen/Strep, was used as culture media and was refreshed every 2–3 days.

PMF isolation

Ear tissues from C57BL/6-J mice, about 3 weeks of age, were clipped into smaller pieces and embedded in 0.2% gelatine in 6-well plates. DMEM supplemented with 20% FCSi and 1% nonessential amino acids (NEAAs; Thermo Fisher Scientific, Waltham, MA, USA, cat. no. 11140050) was added to the embedded ear clippings. After 7 days, skin fibroblasts were grown out of the tissues onto the bottom of the culture plates. PMFs were expanded in culture media up to passage 3. PMFs were then used for further analysis or frozen down and stored in liquid nitrogen for later use.

Primary HUAF isolation

Umbilical cords from full-term pregnancies were collected, stored in sterile PBS at 4°C, and, within 7 days, used for HUAF isolation. The two arteries were isolated from the umbilical cord. Endothelial cells were removed by gently rolling the artery over a blunted needle. After that, the tunica adventitia and tunica media were separated using surgical tools. The tunica adventitia was incubated overnight in culture media supplemented with 10% heat inactivated human serum (PAA, Pasching, Austria) and 1% NEAA. The next day, the tunica adventitia was treated with 2 mg/mL collagenase type II solution (Worthington, OH, USA, cat. no. NC9693955) at 37°C. The resulting cell suspension was filtered over a 70 μm cell strainer and centrifuged at 400 × *g* for 10 min. Cells were plated in 6-well plates, and media were refreshed after 90 min to remove slow adhering nonfibroblast cells. HUAFs were expanded up to passage 3 and used for further analysis or frozen down and stored in liquid nitrogen for later use.

Cellular stress conditions

Oxidative stress in both PMFs and HUAFs was induced by adding 10 μM reactive oxygen species (ROS) mimic tert-butyl hydroperoxide (tBHT; Luperox, 458139, Sigma Aldrich, St. Louis, MO, USA) to the culture media for 24 h. To serum starve the cells, DMEM with 1% PenStrep, supplemented with 1% FCSi for PMFs or 3% FCSi for HUAFs, was added to the cells. Different incubation times were tested for serum starvation (Figure S5). An incubation time of 24 h was determined as the most optimal time point. To induce hypoxia, cells in normal culture media were kept in a humidified incubator at 37°C under 1% O_2 for 24 h. Absolute Ct values of AF357425 and U6 expression are shown in Figure S6.

RNA isolation and qRT-PCR

RNA was isolated by standard TRIzol (Thermo Fisher Scientific, cat. no. 15596026) chloroform extractions. RNA concentration and purity were measured using Nanodrop (Nanodrop Technologies, Wilmington, DE, USA) or the Bioanalyzer (2100 Bioanalyzer Instrument, Agilent, Santa Clara, CA, USA).

Reversed transcription of total RNA was performed with the high-capacity RNA-to-cDNA reverse transcription kit (Applied Biosystems, Thermo Fisher Scientific, cat. no. 4388950). Quantitect SybrGreen reagents (Qiagen Benelux, Venlo, the Netherlands, cat. no. 204145) were used for quantifications. Custom designed TaqMan small RNA assays (Thermo Fisher Scientific, cat. no. 4398987) were used for reversed transcription and quantifications of tRFs. Expression levels were normalized to U6 using the $2^{-\Delta Ct}$ method. All primers used are provided in Table S1.

3GAs and gapmers

3GAs were kindly provided by Idera Pharmaceuticals (Cambridge, MA, USA). 3GAs directed to AF357425 consisted of two identical strands of DNA nucleotides with a full phosphorothioate backbone, connected by a 5′ phosphorothioate linker. GMs were custom designed against AF357425 (GM-AF25) or SNORD113-6 (GM-113; Sigma Aldrich). GMs were made up out of five 2′Ome RNA nucleotides, 10 DNA nucleotides, and five more 2′Ome RNA nucleotides with full phosphorothioate backbone. Sequences of 3GAs and GMs are provided in Table S1.

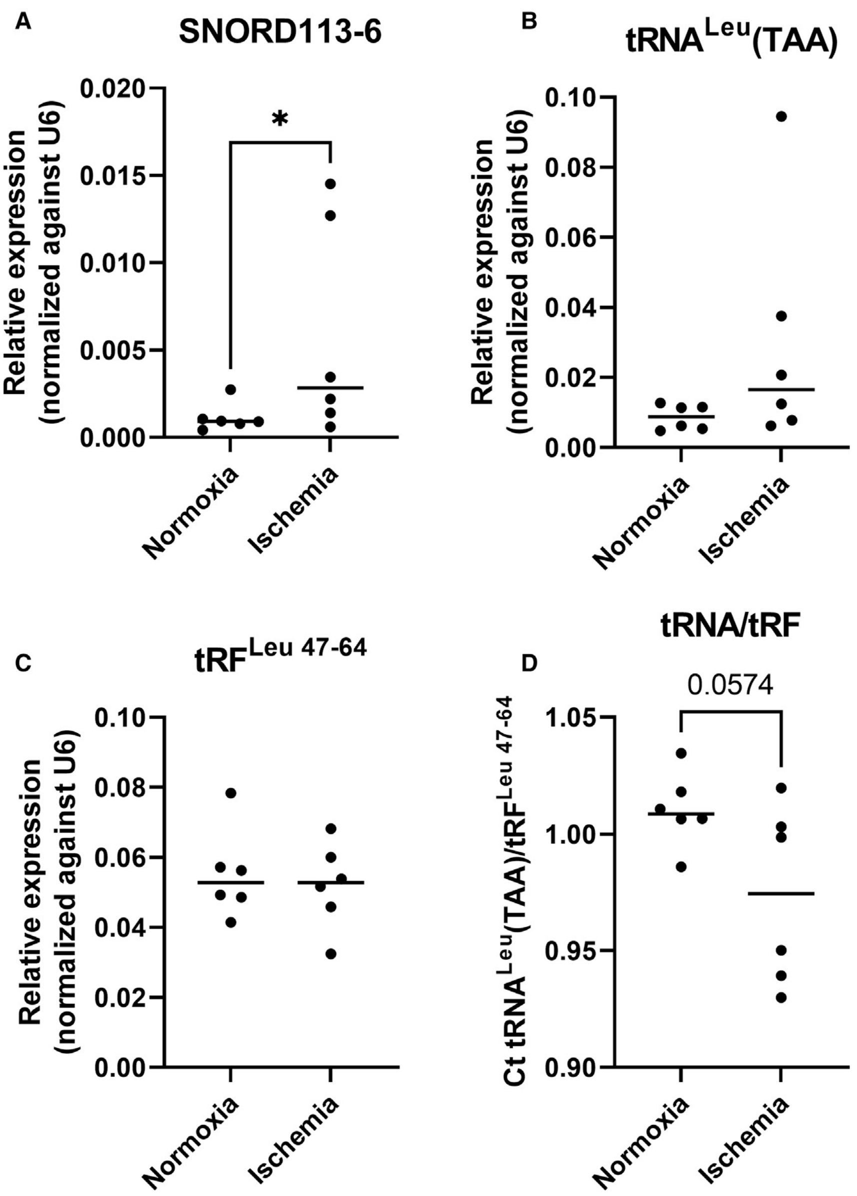

Figure 6. Relative snoRNA, tRNA^{Leu}(TAA), and tRF^{Leu} $_{47–64}$ expression in human inner mammary arteries cultured *ex vivo* under ischemic conditions

Relative expression levels of SNORD113-6 (A), tRNA Leu(TAA) (B), and tRFLeu 47–64 (C), as well as ratios calculated by dividing absolute Ct values of the mature tRNA by Ct values of the tRF (D). n is represented by the individual dots. Data are represented as mean ± SEM. A two-tailed ratio paired t test was performed to compare expression between normoxic and ischemic artery samples. *$p < 0.05$.

Transfection with 3GAs and gapmers

Prior to transfections, G1 cell-cycle arrest was induced by treating cells with KN-93 (Sigma Aldrich, cat. no. K1385), an inhibitor of CaMK-II (the multifunctional Ca^{2+}/CaM kinase). KN-93 was added to the culture media at a concentration of 10 μM for 48 h. After cell synchronization, cells were washed with PBS, and basal DMEM was added. Meanwhile, lipofectamine RNAiMAX Reagent (Thermo Fisher Scientific, cat. no. 13778030) was used to create micelles loaded with 3GAs or GMs against snoRNA AF357425 or SNORD113-6 for transfection. Micelles were added to the cells, and after 1 h of transfection, 10% FCSi was supplemented to the transfected cells. Two concentrations of GM transfection were tested for optimal snoRNA inhibition (Figure S7). The optimal inhibition, without visible cytotoxic effects, was at 500 nM. A timeline of AF357425 and SNORD113-6 inhibition showed efficient snoRNA knockdown at 24 h. Therefore, for all experiments a concentration of GMs (500 nM) or 3GAs (200 nM; as established in a previous study[8]) and a total transfection time of 24 h were used. After 24 h of transfection, cells were washed with PBS and used for further experiments or analyses.

RNA-seq and analysis

RNA was isolated from PMFs transfected with 3GAs or GMs against AF357425. Absolute Ct values of AF357425 and U6 expression are shown in Table S4 and Figure S6. Isolated RNA was shipped to BGI for DNBseq sRNA-seq (GEO: GSE190537). Generated sRNA-seq files in FASTQ format are processed using the sRNA-bench tool.[42] Bowtie aligner was used to align reads to various reference genome and databases, such as GRCm38, mirbase small database, and RNAcentral. The expression of multiple classes of small RNA are quantified in the single assignment-based approach where reads mapping to multiple loci are assigned to the locus that has the highest expression. Reads per million (RPM) normalized counts are further generated that are used for downstream analysis.

Northern blotting

Total RNA samples were diluted in Novex Tris-borate-EDTA (TBE)-urea sample buffer (Thermo Fisher Scientific, cat. no. LC6876), denatured at 95°C for 5 min, and put on ice. 15% Mini-PROTEAN TBE-urea gels (BioRad, cat. no. 4566053) in TBE buffer were pre-run at 200 V for 20 min. After that, RNA samples and a digoxigenin (DIG)-labeled Blue Color Marker for small RNA (DynaMarker, BioDynamics, cat. no. DM270-125uL) were loaded on the gel. Gels were electrophoresed at 200 V for ∼1 h. Next, RNA was transferred from the gel to a Hybond N+ membrane (GE Healthcare, cat. no. RPN203B) at 200 mA for 1 h. A Mini Trans-Blot Electrophoretic Transfer Cell (BioRad) system with an

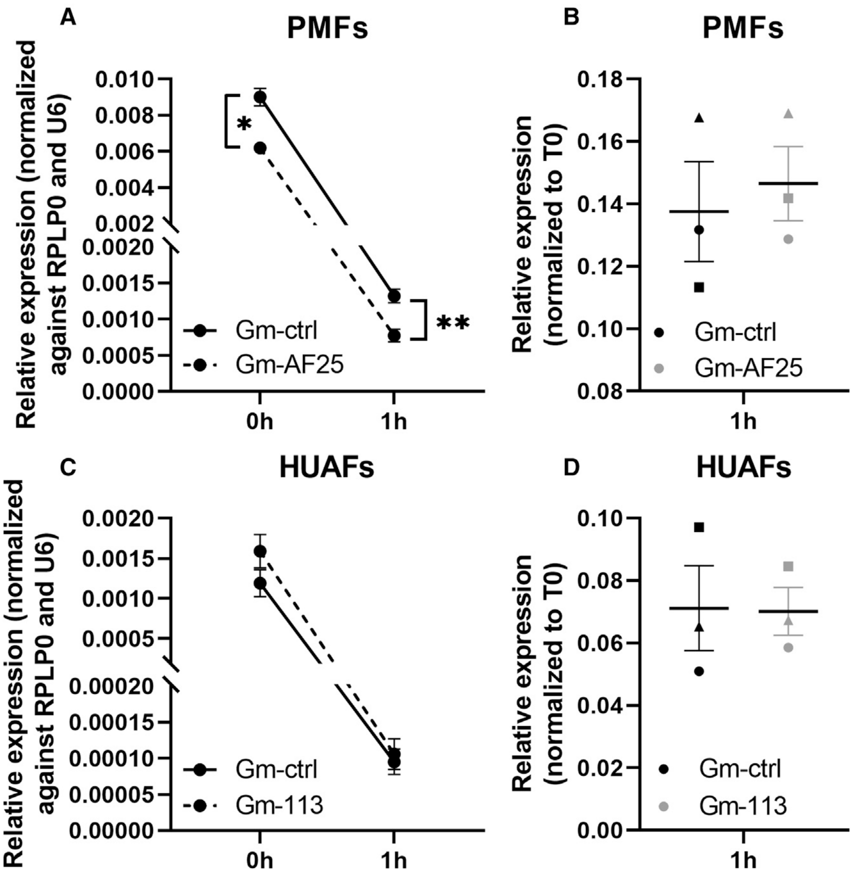

Figure 7. Stability of tRNA^{Leu}(TAA) in primary cells
(A and B) PMFs and (C and D) HUAFs were transfected with GM-AF25/GM-113 or a GM-ctrl for 24 h. After 24 h, cells were treated for 1 h with actinomycin D to inhibit novel RNA transcription. Mature tRNA levels were measured 0 and 1 h after addition of actinomycin D. (A and C) Expression levels were normalized to RPLP0 and U6. n is 3. (B and D) Expression levels at 1 h were normalized to 0 h (T0). n is represented by the individual dots. Data are represented as mean ± SEM. *$p < 0.05$, **$p < 0.01$, compared with GM-ctrl.

ice element and stirrer were used for RNA transfer. Next, RNA was crosslinked to the membrane with freshly prepared 1-ethyl-3-(3-dimethylaminopropyl) carbodiimide (EDC; Sigma, cat. no. E1769) 1-methylimidazole (Sigma, cat. no. 336092) crosslinking solution (pH 8) for 1 h at 60°C. Membranes were pre-hybridized in ULTRAhyb Oligo Hybridization Buffer (Invitrogen, cat. no. AM8663) at 37°C for 30 min while gently shaking. Dual DIG-labelled DNA probes (designed and ordered at Integrated DNA Technologies, Coralville, IA, USA) were denatured at 95°C for 1 min, added to the hybridization buffer (final concentration 5 nM), and left overnight at 37°C. The next day, membranes were washed with low stringency wash buffer (2× SSC, 0.1% SDS) and high stringency wash buffer (0.1× SSC, 0.1% SDS) at 37°C and then washed with 2× SSC buffer at room temperature. Then, membranes were washed and blocked with the DIG Wash and Block Buffer Set (Roche, cat. no. 11585762001) according to the manufacture's protocol. After blocking for 3 h at room temperature while shaking, anti-DIG-AP, Fab fragments (Roche, cat. no. 11093274910) in blocking buffer (1:15.000) were added to the membranes. CDP-star Development Reagent (Roche, cat. no. CDP-RO) was added to the membranes, and images were acquired using ChemiDoc-IT imaging system. Dual DIG-labelled DNA probes are listed in Table S1.

Detection of 2′Ome

For detection of 2′Ome nucleotides, we used an adaptation of the RTL-Q method that was described by Dong et al.[43] To accurately determine the exact location of the 2′Ome site on mature tRNA, a reversed primer downstream of the 2′Ome site (R_D) and a reversed primer on the 2′Ome site (R_U) were designed to the +1 and 0 nt downstream of the predicted 2′Ome nucleotide, respectively. The RT reaction was performed in two consecutive steps. First, a mixture of 20 ng RNA and 10 μM R_D or R_U primers was denatured at 70°C for 5 min and incubated at 42°C for 10 min as an initial annealing step. Then, a high (200 μM) or low (0.5 μM) concentration of dNTPs (Promega, cat. no. U1511), 200 U M-MLV reverse transcriptase (Promega, cat. no. M1705), and 20 U recombinant RNasin ribonuclease inhibitor (Promega, cat. no. N2515) was added to the RT reaction. The RT reaction was incubated at 42°C for 90 min, followed by incubation at 75°C for 15 min. When a 2′Ome site is present, the extension of the R_D primer pauses at this site when low dNTP concentrations are used, whereas the R_U primer does not. Primer extensions are not affected by 2′Ome sites when performing RT at high dNTP concentrations. The differences in RT products were quantified by SYBR green-based qPCR. The EMF was calculated using the following formula.

$$EMF = (Ct\ Low\ dNTP\ R_D - Ct\ High\ dNTP\ R_D) - (Ct\ Low\ dNTP\ R_U - Ct\ High\ dNTP\ R_U)$$

EMF >0 means that methylation is present. EMF ≤ 0 means that no methylation is present.

The sequence of the human precursor tRNA (pre-tRNA) was obtained from publicly available RNA-seq data performed by Gogakos et al.[44] RT primers were designed around the predicted 2′Ome site. One reverse primer was designed upstream of the possible 2′Ome site (R_U) and one downstream of the 2′Ome site (R_D). One forward primer (FW) was used for both R_U and R_D. The same RTL-Q conditions were used as for mature tRNA. All primer sequences are provided in Table S1.

Fibroblast-to-myofibroblast transition

HUAFs were cultured on coverslips and transfected with GM-ctrl or GM-113-6 for 24 h. For collagen staining, cells were fixed in Zinc Formal-Fixx (Thermo Fisher Scientific, cat. no. 6764255) for 30 min and washed with PBS. Collagen was visualized with a picrosirius red staining.

For αSMA staining, cells were fixed in 4% PFA for 15 min and washed in PBS. An antibody against αSMA, 1A4 (Dako M0851, 1:1000), and a secondary antibody Alexa Fluor 555 DαMouse (Invitrogen A31570, 1:1000) were used to visualize αSMA. Hoechst (34580, 1:1,000) was used to stain the nuclei.

Fiji was used to perform immunohistochemistry and immunofluorescence analysis. The area was divided by the total amount of nuclei. The integrated density, which is the sum of values of the pixels, was calculated and divided by the total amount of nuclei (intensity).

Primer sequences for COL1A1 and αSMA (smooth muscle α-2 actin [ACTA2]) are provided in Table S1.

Collection of surplus human artery samples

Human artery samples were collected at the Leiden University Medical Center. Collection, storage, and processing of the samples were performed in compliance with the Medical Treatment Contracts Act (WGBO, 1995) and the Code of Conduct for Health Research using Body Material (Good Practice Code, Dutch Federation of Biomedical Scientific Societies, 2002) and the Dutch Personal Data Protection Act (WBP, 2001).

Human IMAs were harvested during elective coronary bypass surgery from seven patients with coronary artery disease. Only surplus tissue was collected. These samples were anonymized, and no data were recorded that could potentially trace back to an individual's identity. Vessels were left to rest overnight in culture medium (DMEM Glutamax with 10% heat FCSi and 100 U penicillin and 100 μg streptomycin per mL) at 37°C and 20% oxygen and subsequently cultured for 24 h, either at control conditions (20% oxygen and culture medium) or at hypoxia + starvation conditions (1% oxygen and FCS reduced to 0.5%).

Samples were snap frozen and stored at −80°C. Frozen tissues were crushed in liquid nitrogen, and total RNA was isolated from tissue powder by standard TRIzol-chloroform extraction as described above.

Degradation assay

HUAFs and PMFs were treated with KN-93 for 48 h and then transfected with GM-113 or GM-AF25, respectively, or a negative GM-ctrl, as described above. After 24 h of transfection, cells were treated with 5 μg/μL actinomycin D (Sigma Aldrich, cat. no. A9415) to inhibit novel RNA transcription for 1 h. The decline of mature tRNA and the tRF levels over time were quantified by qPCR. Relative AF357425 and SNORD113-6 expression before and after 1 h actinomycin D treatment is shown Figure S8.

Statistical analyses

Results are expressed as mean ± standard error of the mean (SEM). An unpaired t test was performed to compare single treatment with the control. As knockdown efficiency varied per experiment, for these experiments a paired t test was performed to compare each treatment with its own control, within each individual experiment. Graphpad (v.9.0.1) was used to perform all statistical analysis. $p < 0.05$ was considered significant, and $p < 0.1$ was considered a trend.

DATA AND CODE AVAILABILITY

The small RNA-seq data sets of PMFs with overexpression (high) and inhibition (low) of AF357425 were deposited in the Gene Expression Omnibus database under GEO: GSE190537.

SUPPLEMENTAL INFORMATION

Supplemental information can be found online at https://doi.org/10.1016/j.omtn.2022.09.011.

ACKNOWLEDGMENTS

The study was supported by funding from the Rembrandt Institute of Cardiovascular Science and the Austrian Science Fund (FWF; Lise Meitner grant M2578-B30) and the Netherlands Organisation for Scientific Research (NWO; Women in STEM 2021–18992). We thank Dr. Jesper Hjortnaes and Dr. Roemer J. Vos for the collection of human arterial tissue samples and Julia C.W. van Lent for technical assistance.

AUTHOR CONTRIBUTIONS

E.v.I., P.H.A.Q., and A.Y.N. designed the experiments; E.v.I., T.W., P.A.M.E., M.L.v.d.B., and H.M. conducted the experiments; E.v.I., J.W., P.H.A.Q., and A.Y.N. wrote, reviewed, and edited the paper; A.Y.N. acquired funding; A.Y.N. and P.H.A.Q. supervised.

DECLARATION OF INTERESTS

There are no conflicts of interest.

REFERENCES

1. Gibbons, G.H., and Dzau, V.J. (1994). The emerging concept of vascular remodeling. N. Engl. J. Med. *330*, 1431–1438.
2. Tinajero, M.G., and Gotlieb, A.I. (2020). Recent developments in vascular adventitial pathobiology: the dynamic adventitia as a complex regulator of vascular disease. Am. J. Pathol. *190*, 520–534.
3. Esteller, M., and Pandolfi, P.P. (2017). The epitranscriptome of noncoding RNAs in cancer. Cancer Discov. *7*, 359–368.
4. Hoernes, T.P., and Erlacher, M.D. (2017). Translating the epitranscriptome. WIREs RNA *8*. https://doi.org/10.1002/wrna.1375.
5. Pinto, Y., Buchumenski, I., Levanon, E.Y., and Eisenberg, E. (2018). Human cancer tissues exhibit reduced A-to-I editing of miRNAs coupled with elevated editing of their targets. Nucleic Acids Res. *46*, 71–82.
6. Riemondy, K.A., Gillen, A.E., White, E.A., Bogren, L.K., Hesselberth, J.R., and Martin, S.L. (2018). Dynamic temperature-sensitive A-to-I RNA editing in the brain of a heterothermic mammal during hibernation. Rna *24*, 1481–1495.
7. van der Kwast, R.V.C.T., van Ingen, E., Parma, L., Peters, H.A.B., Quax, P.H.A., and Nossent, A.Y. (2018). Adenosine-to-Inosine editing of MicroRNA-487b alters target gene selection after ischemia and promotes neovascularization. Circ. Res. *122*, 444–456.

8. Håkansson, K.E.J., Goossens, E.A.C., Trompet, S., van Ingen, E., de Vries, M.R., van der Kwast, R.V.C.T., Ripa, R.S., Kastrup, J., Hohensinner, P.J., Kaun, C., et al. (2019). Genetic associations and regulation of expression indicate an independent role for 14q32 snoRNAs in human cardiovascular disease. Cardiovasc. Res. *115*, 1519–1532.

9. Håkansson, K.E.J., Sollie, O., Simons, K.H., Quax, P.H.A., Jensen, J., and Nossent, A.Y. (2018). Circulating small non-coding RNAs as biomarkers for recovery after exhaustive or repetitive exercise. Front. Physiol. *9*, 1136.

10. Nossent, A.Y., Ektefaie, N., Wojta, J., Eichelberger, B., Kopp, C., Panzer, S., and Gremmel, T. (2019). Plasma levels of snoRNAs are associated with platelet activation in patients with peripheral artery disease. Int. J. Mol. Sci. *20*, E5975.

11. Civitarese, R.A., Kapus, A., McCulloch, C.A., and Connelly, K.A. (2017). Role of integrins in mediating cardiac fibroblast-cardiomyocyte cross talk: a dynamic relationship in cardiac biology and pathophysiology. Basic Res. Cardiol. *112*, 6.

12. Ingen, E., Homberg, D.A.L., Bent, M.L., Mei, H., Papac-Milicevic, N., Kremer, V., Boon, R.A., Quax, P.H.A., Wojta, J., and Nossent, A.Y. (2021). C/D box snoRNA SNORD113-6/AF357425 plays a dual role in integrin signalling and arterial fibroblast function via pre-mRNA processing and 2′O-ribose methylation. Hum. Mol. Genet. https://doi.org/10.1093/hmg/ddab304.

13. Kiss, T. (2001). Small nucleolar RNA-guided post-transcriptional modification of cellular RNAs. The EMBO journal *20*, 3617–3622.

14. Ojha, S., Malla, S., and Lyons, S.M. (2020). snoRNPs: functions in ribosome biogenesis. Biomolecules *10*, E783.

15. Watkins, N.J., and Bohnsack, M.T. (2012). The Box C/D and H/ACA snoRNPs: Key players in the modification, processing and the dynamic folding of ribosomal RNA. WIREs RNA *3*, 397–414.

16. Jorjani, H., Kehr, S., Jedlinski, D.J., Gumienny, R., Hertel, J., Stadler, P.F., Zavolan, M., and Gruber, A.R. (2016). An updated human snoRNAome. Nucleic Acids Res. *44*, 5068–5082.

17. Falaleeva, M., Welden, J.R., Duncan, M.J., and Stamm, S. (2017). C/D-box snoRNAs form methylating and non-methylating ribonucleoprotein complexes: old dogs show new tricks. Bioessays *39*, 1600264.

18. Elliott, B.A., Ho, H.T., Ranganathan, S.V., Vangaveti, S., Ilkayeva, O., Abou Assi, H., Choi, A.K., Agris, P.F., and Holley, C.L. (2019). Modification of messenger RNA by 2′-O-methylation regulates gene expression *in vivo*. Nat. Commun. *10*, 3401.

19. Nostramo, R.T., and Hopper, A.K. (2019). Beyond rRNA and snRNA: tRNA as a 2′-O-methylation target for nucleolar and Cajal body box C/D RNPs. Genes Dev. *33*, 739–740.

20. Pereira, M., Francisco, S., Varanda, A.S., Santos, M., Santos, M.A.S., and Soares, A.R. (2018). Impact of tRNA modifications and tRNA-modifying enzymes on proteostasis and human disease. Int. J. Mol. Sci. *19*, E3738.

21. Krishna, S., Raghavan, S., DasGupta, R., and Palakodeti, D. (2021). tRNA-derived fragments (tRFs): establishing their turf in post-transcriptional gene regulation. Cell. Mol. Life Sci. *78*, 2607–2619.

22. Keam, S.P., and Hutvagner, G. (2015). tRNA-derived fragments (tRFs): emerging new roles for an ancient RNA in the regulation of gene expression. Life *5*, 1638–1651.

23. Fu, H., Feng, J., Liu, Q., Sun, F., Tie, Y., Zhu, J., Xing, R., Sun, Z., and Zheng, X. (2009). Stress induces tRNA cleavage by angiogenin in mammalian cells. FEBS Lett. *583*, 437–442.

24. Li, Q., Hu, B., Hu, G.W., Chen, C.Y., Niu, X., Liu, J., Zhou, S.M., Zhang, C.Q., Wang, Y., and Deng, Z.F. (2016). tRNA-derived small non-coding RNAs in response to ischemia inhibit angiogenesis. Sci. Rep. *6*, 20850.

25. Elkordy, A., Mishima, E., Niizuma, K., Akiyama, Y., Fujimura, M., Tominaga, T., and Abe, T. (2018). Stress-induced tRNA cleavage and tiRNA generation in rat neuronal PC12 cells. J. Neurochem. *146*, 560–569.

26. Vitali, P., and Kiss, T. (2019). Cooperative 2′-O-methylation of the wobble cytidine of human elongator tRNA(Met)(CAT) by a nucleolar and a Cajal body-specific box C/D RNP. Genes Dev. *33*, 741–746.

27. Guzzi, N., Cieśla, M., Ngoc, P.C.T., Lang, S., Arora, S., Dimitriou, M., Pimková, K., Sommarin, M.N.E., Munita, R., Lubas, M., et al. (2018). Pseudouridylation of tRNA-derived fragments steers translational control in stem cells. Cell *173*, 1204–1216.e26.

28. Lyons, S.M., Fay, M.M., and Ivanov, P. (2018). The role of RNA modifications in the regulation of tRNA cleavage. FEBS Lett. *592*, 2828–2844.

29. Clouet d'Orval, B., Bortolin, M.L., Gaspin, C., and Bachellerie, J.P. (2001). Box C/D RNA guides for the ribose methylation of archaeal tRNAs. The tRNATrp intron guides the formation of two ribose-methylated nucleosides in the mature tRNATrp. Nucleic Acids Res. *29*, 4518–4529.

30. van der Kwast, R., Parma, L., van der Bent, M.L., van Ingen, E., Baganha, F., Peters, H.A.B., Goossens, E.A.C., Simons, K.H., Palmen, M., de Vries, M.R., et al. (2020). Adenosine-to-Inosine editing of vasoactive MicroRNAs alters their targetome and function in ischemia. Mol. Ther. Nucleic Acids *21*, 932–953.

31. GtRNAdb tRNAscan-SE Analysis of Complete GenomesJune 2021

32. Motorin, Y., and Helm, M. (2010). tRNA stabilization by modified nucleotides. Biochemistry *49*, 4934–4944.

33. van Ingen, E., van den Homberg, D.A.L., van der Bent, M.L., Mei, H., Papac-Milicevic, N., Kremer, V., Boon, R.A., Quax, P.H.A., Wojta, J., and Nossent, A.Y. (2022). C/D box snoRNA SNORD113-6/AF357425 plays a dual role in integrin signalling and arterial fibroblast function via pre-mRNA processing and 2′O-ribose methylation. Hum. Mol. Genet. *31*, 1051–1066.

34. Yamasaki, S., Ivanov, P., Hu, G.F., and Anderson, P. (2009). Angiogenin cleaves tRNA and promotes stress-induced translational repression. J. Cell Biol. *185*, 35–42.

35. Nguyen, T.T.M., van der Bent, M.L., Wermer, M.J.H., van den Wijngaard, I.R., van Zwet, E.W., de Groot, B., Quax, P.H.A., Kruyt, N.D., and Nossent, A.Y. (2020). Circulating tRNA fragments as a novel biomarker class to distinguish acute stroke subtypes. Int. J. Mol. Sci. *22*, E135.

36. Ishida, T., Inoue, T., Niizuma, K., Konno, N., Suzuki, C., Inoue, T., Ezura, M., Uenohara, H., Abe, T., and Tominaga, T. (2020). Prediction of functional outcome in patients with acute stroke by measuring tRNA derivatives. Cerebrovasc. Dis. *49*, 639–646.

37. Magee, R., Londin, E., and Rigoutsos, I. (2019). TRNA-derived fragments as sex-dependent circulating candidate biomarkers for Parkinson's disease. Park. Relat. Disord. *65*, 203–209.

38. Su, Z., Wilson, B., Kumar, P., and Dutta, A. (2020). Noncanonical roles of tRNAs: tRNA fragments and beyond. Annu. Rev. Genet. *54*, 47–69.

39. Magee, R., and Rigoutsos, I. (2020). On the expanding roles of tRNA fragments in modulating cell behavior. Nucleic Acids Res. *48*, 9433–9448.

40. Liapi, E., van Bilsen, M., Verjans, R., and Schroen, B. (2020). tRNAs and tRNA fragments as modulators of cardiac and skeletal muscle function. Biochim. Biophys. Acta. Mol. Cell Res. *1867*, 118465.

41. Potapov, V., Fu, X., Dai, N., Corrêa, I.R., Jr., Tanner, N.A., and Ong, J.L. (2018). Base modifications affecting RNA polymerase and reverse transcriptase fidelity. Nucleic Acids Res. *46*, 5753–5763.

42. Aparicio-Puerta, E., Lebrón, R., Rueda, A., Gómez-Martín, C., Giannoukakos, S., Jaspez, D., Medina, J.M., Zubkovic, A., Jurak, I., Fromm, B., et al. (2019). sRNAbench and sRNAtoolbox 2019: intuitive fast small RNA profiling and differential expression. Nucleic Acids Res. *47*. W530–w535.

43. Dong, Z.W., Shao, P., Diao, L.T., Zhou, H., Yu, C.H., and Qu, L.H. (2012). RTL-P: a sensitive approach for detecting sites of 2'-O-methylation in RNA molecules.Nucleic. Acids Res *40*, e157. https://doi.org/10.1093/nar/gks698.

44. Gogakos, T., Brown, M., Garzia, A., Meyer, C., Hafner, M., and Tuschl, T. (2017). Characterizing expression and processing of precursor and mature human tRNAs by hydro-tRNAseq and PAR-CLIP. Cell Rep. *20*, 1463–1475.

OMTN, Volume *30*

Supplemental information

C/D box snoRNA SNORD113-6 guides 2′-O-methylation and protects against site-specific fragmentation of tRNA^{Leu}(TAA) in vascular remodeling

Eva van Ingen, Pleun A.M. Engbers, Tamar Woudenberg, M. Leontien van der Bent, Hailiang Mei, Johann Wojta, Paul H.A. Quax, and A. Yaël Nossent

Supplemental Tables

Table S1. Oligonucleotide and primer sequences. 3rd Generation Antisense (3GA) and gapmers (GM). $[nnn]_D$ = DNA-nucleotides; $[nnn]_{2'OmeR}$ = 2'Ome-RNA nucleotides; X = phosphorothioate linker.

**Oligonucleotide**	
3GA-AF357425	3'-$[GGGTTTAATCACTGTCCTC]_D$-X-$[CTCCTGTCACTAATTTGGG]_D$-3'
GM-AF357425-D'	3'-$[GUCAG]_{2'OmeR}[AAACCCCATG]_D[CUCCU]_{2'OmeR}$-5'
GM-113-6-D'	3'-$[CAGAA]_{2'OmeR}[ACCCCATGAT]_D[ATTCA]_{2'OmeR}$-5'
GM-Control	3'-$[AUCGA]_{2'OmeR}[TACCGTATAA]_D[UAACG]_{2'OmeR}$-5'
**Northern blot**	
Dual DIG-labelled DNA probes	/5Dig/ACCCACGCAGACATATGT/3Dig/
**Methylation primers**	
Mature $tRNA^{Leu}$(TAA) HSA/MMU FW	CCGAGTGGTTAAGGCGTTG
Mature $tRNA^{Leu}$(TAA) HSA/MMU R_D	TGGTACCAGGAGTGG
Mature $tRNA^{Leu}$(TAA) HSA/MMU R_U	GGTACCAGGAGTGGG
pre-$tRNA^{Leu}$(TAA) HSA FW	AAACAAGGTTCAACGTCTGCA
pre-$tRNA^{Leu}$(TAA) HSA R_D	ACCTAAAGCTACCAGGAGTGG
pre-$tRNA^{Leu}$(TAA) HSA R_U	CGAACCCACGCGGACATATG
**qPCR primers**	
Mature $tRNA^{Leu}$(TAA) FW	CCGAGTGGTTAAGGCGTTG
Mature $tRNA^{Leu}$(TAA) RV	CAGGAGTGGGGTTCGAAC
**Taqman Custom designed**	
tRF^{47-64} MMU	UGGACAUAUGUCUGCGUGGGU
tRF^{47-64} HSA	UGGACAUAUGUCCGCGUGGGU
**Housekeeping genes**	
U6– MMU/HSA – FW	AGAAGATTAGCATGGCCCCT
U6– MMU/HSA – RV	ATTTGCGTGTCATCCTTGCG
RPLP0 HSA FW	TCCTCGTGGAAGTGACATCG
RPLP0 HSA RV	TGTCTGCTCCCACAATGAAAC
RPLP0 MMU FW	GTGATGCCCAGGGAAGACAG

RPLP0 MMU RV	TCTGCTCCCACAATGAAGCA
**SnoRNAs**	
SNORD113-6 – FW	TGGACCAGTGATGAATATCATG
SNORD113-6 – RV	TGGACCTCAGAGTTGCAGATG
AF357425 – FW	AGGAGCATGGGGTTTCTGAC
AF357425 – RV	TTTCATAAGGGTTTAATCACTGTCC
**Angiogenin**	
HSA FW	CTGGGCGTTTTGTTGTTGGTC
HSA RV	GGTTTGGCATCATAGTGCTGG
MMU FW	CCAGGCCCGTTGTTCTTGAT
MMU RV	GCAAACCATTCTCACAGGCAATA
**COL1A1**	
HSA FW	TGTGATGGGATTCCCTGGACCTAAAG
HSA RV	TGAGCTCCAGCCTCTCCATCTTTG
**ACTA2**	
HSA FW	AGATCCTGACTGAGCGTGGCTATTC
HSA RV	CTCTTCTCAAGGGAGGATGAGGATGC

Table S2. Expression of tRNA fragments in reads per million (RPM). Small RNA sequencing was performed on primary murine fibroblasts with AF357425 overexpression (High) and knockdown (Low). Fold change is calculated by dividing High by Low RPM. Read counts are quantified in the single assignment approach where reads that map to multiple loci are assigned to the locus that has the highest expression.

antiCodon	High RPM	Low RPM	FoldChange
AlaAGC	342	317	1,08
AlaCGC	169	198	0,85
AlaTGC	334	387	0,86
ArgACG	686	338	2,03
ArgCCG	304	188	1,61
ArgCCT	1000	637	1,57
ArgTCG	786	376	2,09
ArgTCT	235	227	1,04
AsnGTT	446	101	4,40
AspGTC	1109	628	1,77
CysGCA	159	106	1,50
GlnCTG	144	150	0,96
GlnTTG	85	79	1,07
GluCTC	1153	735	1,57
GluTTC	640	489	1,31
GlyACC	22	13	1,67
GlyCCC	167	179	0,94
GlyGCC	817	806	1,01
GlyTCC	322	445	0,72
HisATG	2	2	1,09
HisGTG	213	160	1,33
IleAAT	957	811	1,18
IleGAT	11	7	1,66
IleTAT	20	16	1,20
LeuAAG	256	382	0,67
LeuCAA	255	178	1,43
LeuCAG	272	221	1,23
LeuTAA	238	121	1,97
LeuTAG	157	217	0,72
LysCTT	1758	1357	1,30
LysTTT	1241	1156	1,07
MetCAT	984	437	2,25
PheGAA	250	99	2,53
ProAGG	853	347	2,46

ProCGG	726	355	2,04
ProTGG	2659	1319	2,02
SeC(e)TCA	16	7	2,19
SeCTCA	1	1	0,67
SerAGA	470	406	1,16
SerCGA	77	44	1,75
SerGCT	139	74	1,88
SerGGA	25	8	2,95
SerTGA	161	144	1,12
SupTTA	3	1	2,42
ThrAGT	203	192	1,06
ThrCGT	148	87	1,69
ThrTGT	280	188	1,49
TrpCCA	333	245	1,36
TyrGTA	1610	736	2,19
ValAAC	233	178	1,30
ValCAC	328	261	1,26
ValTAC	88	85	1,04

Table S3. Read counts small RNA sequencing. Small RNA sequencing was performed on PMFs with overexpression (High) or inhibition (Low) of AF357425.

	**High AF357425**	**Low AF357425**
miRbase (sense)	946057,48	951864,44
tRNA	24527,6	16588,28
snoRNA	3167,57	3262,12
Cdna (sense)	2620,37	2459,04
rRNA	2138,41	2084,58
mRNA (antisense)	911,45	845,88
snRNA	96,46	99,15
piRNA	51,18	33,09

Table S4. Absolute Ct values of AF357425 and U6. Small RNA sequencing was performed on primary murine fibroblasts with AF357425 overexpression (3GA-AF25) and knockdown (GM-AF25).

**Sample**	**Ct AF357425**	**Ct U6**
3GA-AF25	21,58	20,65667
GM-AF25	23,71	20,39667

Supplemental Figures

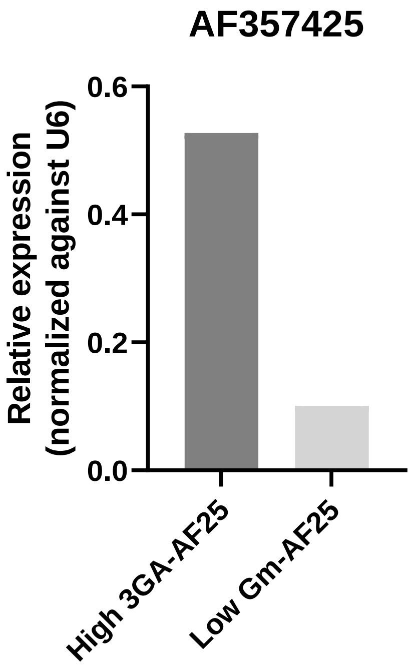

Figure S1. Relative AF357425 expression in small RNA-seq samples. Primary murine fibroblasts were transfected with 3rd Generation Antisense (High 3GA-AF25) or Gapmers (Low Gm-AF25) against AF357425 for 24 hours. Expression levels are normalized to U6.

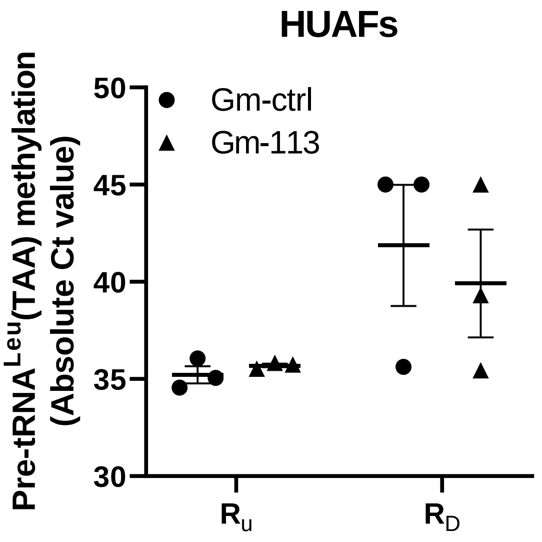

Figure S2. Precursor (pre-)tRNA^{Leu}(TAA) methylation in human arterial fibroblasts (HUAFs). For detection of 2'O-methyated nucleotides, Reversed Transcription at Low dNTP concentration followed by Quantitative PCR (RTL-Q) was performed. Reversed primers were designed upstream (R_U) and downstream (R_D) of the predicted 2'O-methylation (2'Ome) site. RT was performed at low dNTP concentrations. When a 2'Ome site is present, the extension of the R_D primer pauses at this site when low dNTP concentrations are used, whereas the R_U primer does not. Results are shown in absolute Ct values. Ct values above the detection threshold are shown as 45 Ct. N is represented by the individual dots.

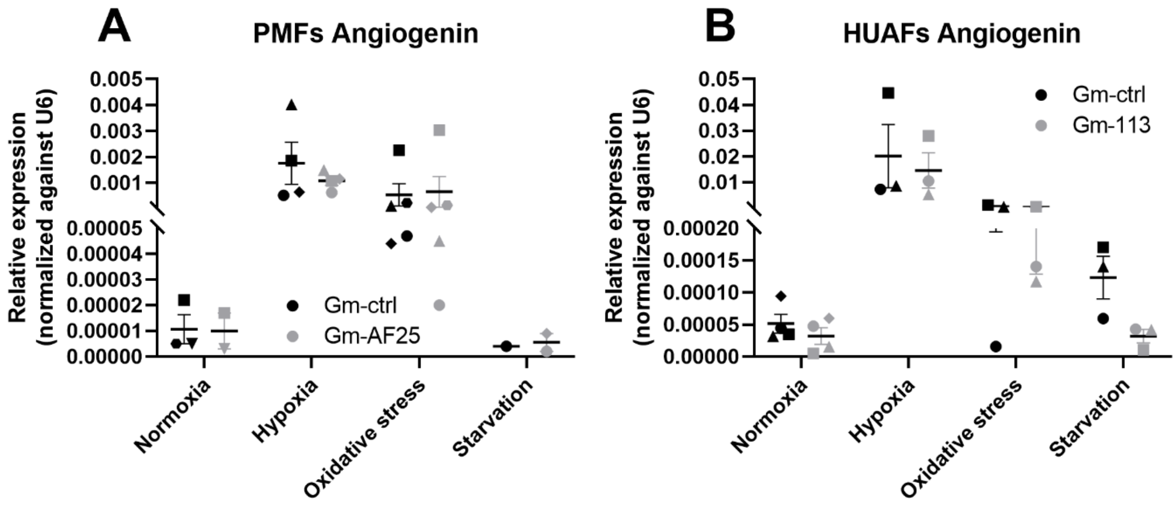

Figure S3. Angiogenin expression in primary cells during cellular stress. (A) Primary murine fibroblasts (PMFs) and (B) human umbilical arterial fibroblasts (HUAFs) were transfected with either Gm-ctrl or Gm-AF25/113 and cultured in normoxic (control), hypoxic, oxidative stress or starvation conditions for 24h. Relative expression levels are normalized to U6. N is represented by the individual dots.

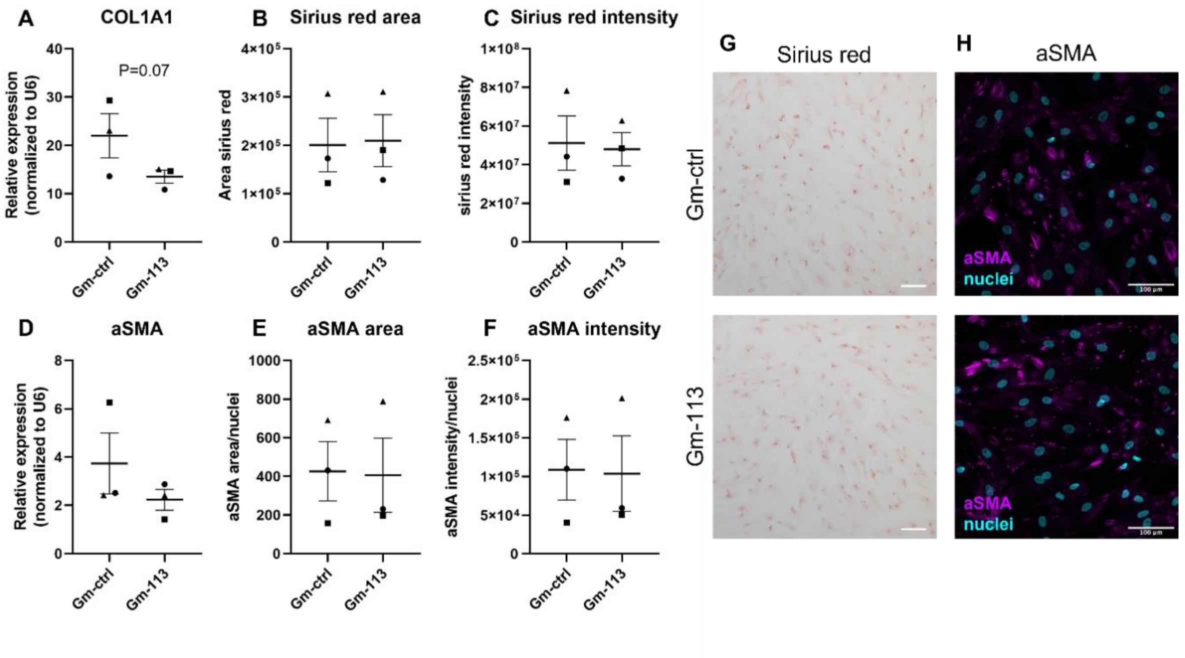

Figure S4. Collagen and alpha-smooth muscle actin (aSMA) expression in human umbilical arterial fibroblasts (HUAFs). (A) Relative expression collagen type 1 alpha 1 chain, (B) area and (C) intensity of Sirius red staining, (D) relative expression of aSMA, (E) area and (F) intensity of aSMA, representative images of (G) Sirius red and (H) aSMA staining of Gm-ctrl and Gm-113 treated HUAFs. HUAFs were treated with Gm-ctrl or Gm-113-6 for 24h. (A, D) Expression levels were normalized to U6. (B,C,D-H) The area was divided by the total amount of nuclei. The integrated density, which is the sum of values of the pixels, was calculated and divided by the total amount of nuclei (intensity). N is represented by the individual dots.

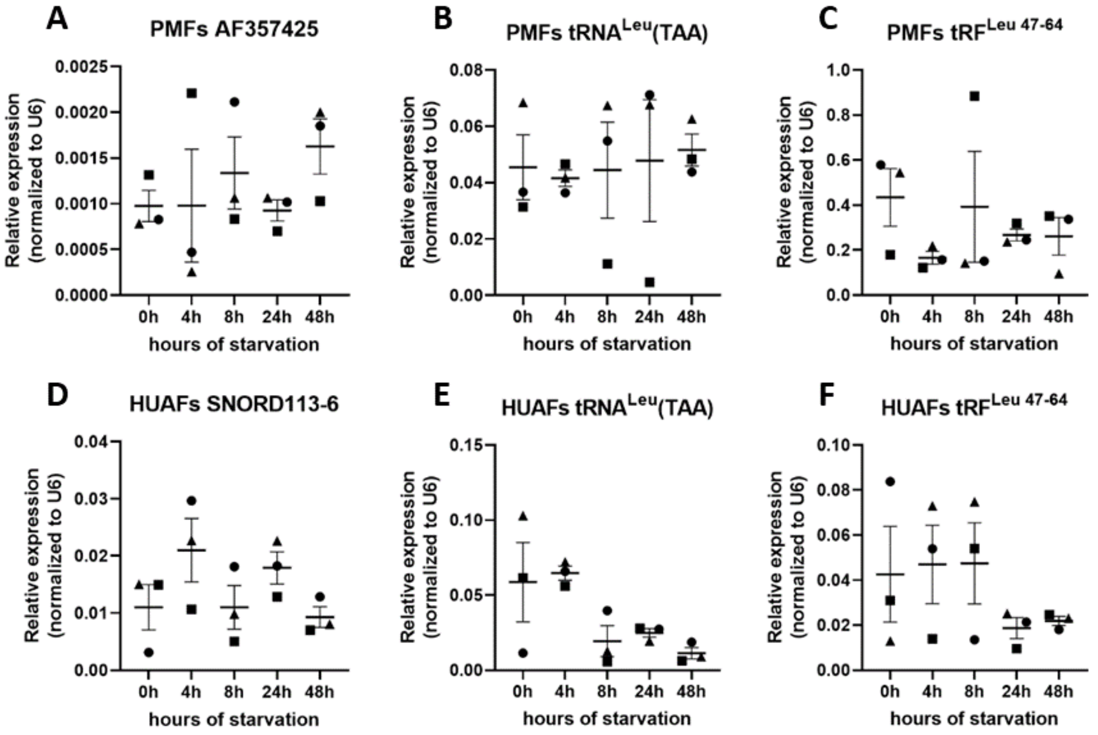

Figure S5. Primary murine fibroblasts (PMFs) and human umbilical arterial fibroblasts (HUAFs) were cultured under different times of serum starvation. Relative expression of (A) AF35742525, (B) tRNA Leucine anti-codon TAA (tRNA^{Leu}(TAA)) and tRNA fragment tRF$^{Leu\ 47\text{-}64}$ in PMFs. Relative expression of (D) SNORD113-6, (E) tRNA^{Leu}(TAA) and (F) tRF$^{Leu\ 47\text{-}64}$ in HUAFs. N is represented by the individual dots.

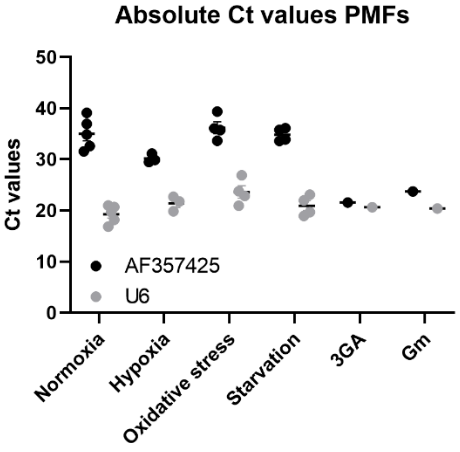

Figure S6. Absolute Ct values of AF357425 and U6 expression in primary murine fibroblasts (PMFs) cultured under different stress conditions or transfected with 3rd Generation Antisense (3GA) or GM for small RNA sequencing. PMFs were cultured under normoxia, hypoxia, oxidative stress or starvation for 24h. Transfection for small RNA sequencing was performed with either 3GA (200 nM) or GM (500 nM) for 24h. N is represented by the individual dots.

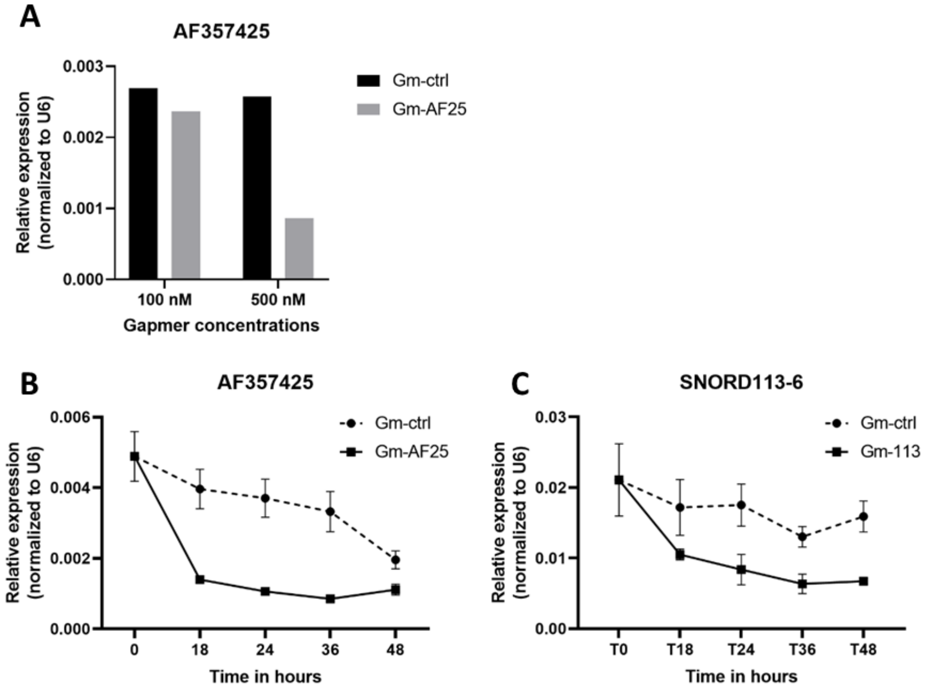

Figure S7. Optimization of GM transfection. Two concentrations of GM and different times of transfection were tested for optimal inhibition. (A) Primary murine fibroblasts (PMFs) were transfected with a concentration of 100 or 500 nM GM against AF357425 (GM-AF25) or a control (GM-ctrl) for

24h. Different times of transfection against (B) AF357425 in PMFs and (C) SNORD113-6 in HUAFs were tested (N=3). (B,C) Relative expression levels are normalized to U6.

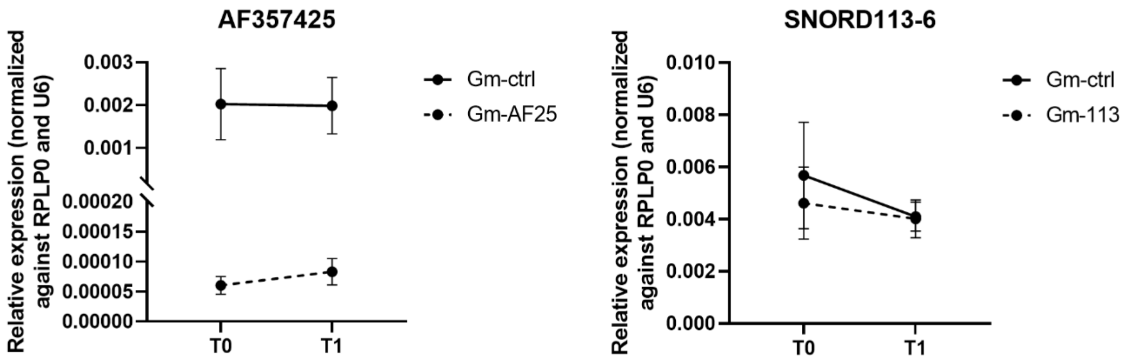

Figure S8. Relative AF357425 and SNORD113-6 expression in PMFs and HUAFs, respectively, before and after 1h Actinomycin D treatment. Cells were first transfected with GM against AF357425 (Gm-AF25) or SNORD113-6 (Gm-113), or a control (Gm-ctrl), and then treated with Actinomycin D. Expression levels are normalized to RPLP0 and U6.
